# Emodin Enhanced Microwave‐Responsive Heterojunction with Powerful Bactericidal Capacity and Immunoregulation for Curing Bacteria‐Infected Osteomyelitis

**DOI:** 10.1002/advs.202409979

**Published:** 2024-11-27

**Authors:** Tao Xu, Hao Cheng, Hailiang Pei, Jiameng Wang, Yiwei Shi, Xiangyu Zhang, Di Huang

**Affiliations:** ^1^ Department of Biomedical Engineering Research Center for Nano‐biomaterials & Regenerative Medicine College of Artificial Intelligence Taiyuan University of Technology Taiyuan 030024 China; ^2^ Shanxi Key Laboratory of Biomedical Metal Materials College of Materials Science and Engineering Taiyuan University of Technology Taiyuan 030024 China; ^3^ NHC Key Laboratory of Pneumoconiosis Department of Pulmonary and Critical Care Medicine First Hospital of Shanxi Medical University Taiyuan 030001 China

**Keywords:** antimicrobial, dielectric losses, heterogeneous interfaces, microwave response, osteomyelitis

## Abstract

Eradication of osteomyelitis caused by bacterial infections is still a major challenge. Microwave therapy has the inherent advantage of deep penetration in curing deep tissue infections. However, the antibacterial efficiency of sensitizers is limited by the weak energy of microwaves. Here, a hybrid heterojunction system (Fe_3_O_4_/CuS/Emo) is designed for curing bacterially infected osteomyelitis. As an enhanced microwave sensitizer, it shows supernormal microwave response ability. Specifically, Fe_3_O_4_ acts as a matrix to mediate magnetic loss. After CuS loading, the heterogeneous interface forms induce significant interfacial polarization, which increasing dielectric loss. On the basis of the heterojunction formed by the two semiconductors, emodin is innovatively introduced to modify it. This integration not only accelerates the movement of charge carriers but also enhances polarization loss due to the numerous functional groups present on the surface. This further optimizes the microwave thermal and catalytic response. In addition, the unique anti‐inflammatory properties of emodin confer the ability of hybrid heterojunction to regulate the immune microenvironment. In vivo studies reveal that heterojunction modified by emodin programmed elimination of bacteria and regulation of the immune microenvironment. It offers a revolutionary approach to the treatment of bacterial osteomyelitis.

## Graphical abstract

This study designs a magnetic heterojunction with enhanced microwave response. It can generate ROS and heat through microwave response, thereby killing bacteria. In addition, the introduction of emodin is conducive for remodeling of the immune microenvironment and bone regeneration. This is of great value for improving the therapeutic effect through the dual effects of effective sterilization and anti‐inflammation.

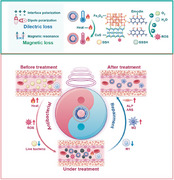

## 1 Introduction

Osteomyelitis is a common, purulent microbial infection that induces inflammation in various bone tissues (periosteum, trabecular bone, bone marrow, etc.).^[^ ^1^ ^]^ Addressing bacterial infection is fundamental to the treatment of osteomyelitis. However, due to the unique physiological characteristics of bone, osteomyelitis often affects deep tissue layers and complicates the treatment process.^[^ ^2^ ^]^ The common treatment is debridement coupled with systemic antibiotic therapy.^[^ ^3^ ^]^ However, with the widespread evolution of bacterial resistance brought on by antibiotic abuse, the effectiveness of the treatment has been significantly diminished.^[^ ^3^, ^4^ ^]^ In abundant effective antibiotic‐free treatments, light‐assisted antibacterial therapy is developing most rapidly due to the characteristics of exceptional spatiotemporal selectivity.^[^ ^5^ ^]^ However, its inadequate tissue penetration ability makes it unsuitable for managing deep bone tissue infections.

In contrast to light‐assisted therapy, microwave (MW) is believed to have fewer adverse effects and enhanced penetration, positioning it as a promising adjunctive option for osteomyelitis management.^[^ ^6^ ^]^ Certain microwave‐sensitive agents (TiO_2_, liquid metals, Zr MOFs, etc.) can quickly attain elevated temperatures under microwave irradiation.^[^ ^7^ ^]^ Unfortunately, the effectiveness of microwave thermotherapy (MWT) may be hampered by bacterial resistance to high heat,^[^ ^8^ ^]^ and excessive temperature can harm healthy cells. Consequently, MWT predominantly operates in tandem with microwave dynamic therapy (MDT) which has the potential to generate storms of reactive oxygen species (ROS).^[^ ^9^ ^]^ Nevertheless, single‐component materials often fall short in terms of effective microwave absorption (MA) and the catalytic ability to produce ROS, which inhibits a seamless integration of MWT and MDT. Thus, promoting coordination between multiple components of materials to improve microwave response performance is a key factor in upgrading antimicrobial strategies supported by nanomaterials.

In order to realize effective integration and cooperation between MWT and MDT, the MA and ROS catalytic ability are usually enhanced by the formation of oxygen vacancies, oxygen defects, Fenton reactions, and Fenton‐like reactions.^[^ ^6^, ^10^ ^]^ One prominent approach involves creating a heterojunction, which comprises a heterogeneous interface formed by two semiconductors that possess varying energy levels and band structures. The electron interactions across these disparate energy bands are significant, leading to a redistribution of the internal electric field and resulting in remarkable catalytic efficiency. Consequently, there is substantial potential for creating nanomaterials with outstanding microwave response capabilities in the biomedical sector through the engineering of interface heterojunctions.

The heterogeneous interface created through the interplay of dielectric loss and magnetic loss components can lead to enhanced interface loss and impedance matching, which will be more conducive to MA and catalytic properties.^[^ ^10^, ^11^ ^]^ Ferrosoferric oxide (Fe_3_O_4_) is a highly sought‐after magnetic loss material, thanks to its intrinsic ferromagnetic resonance and stability. Additionally, it has received FDA approval for medical applications due to its exceptional biocompatibility.^[^ ^12^ ^]^ However, since Fe_3_O_4_ lacks robust antibacterial efficacy on its own, it is necessary to pair it with an additional dielectric material that can provide effective antibacterial support. Copper(II) sulfide (CuS) is a classical dielectric loss material known for the remarkable antibacterial properties and good biodegradability.^[^ ^13^ ^]^ Additionally, the ongoing bacterial infection in the osteomyelitis environment triggers a robust immune response, leading to significant bone damage.^[^ ^14^ ^]^ Therefore, remodeling of the immune microenvironment is also indispensable in the later stage of treatment.^[^ ^15^ ^]^ Emodin exhibits several biological properties, including the inhibition of oxidative stress, anti‐inflammatory, antimicrobial, and promote bone regeneration in some studies.^[^ ^16^ ^]^ More importantly, emodin acts as a multivalent molecule presenting a *π*‐conjugated structure. Its distinctive architecture aids in generating additional charge carriers and enhances their mobility.^[^ ^17^ ^]^ Moreover, the abundant active groups on the surface further attenuated the microwave through the enhancement of dipole polarization,^[^ ^18^ ^]^ which would further optimize the MA. Therefore, their integration presents an excellent opportunity for creating heterojunction materials that exhibit exceptional responsiveness to microwaves.

**Scheme 1:**
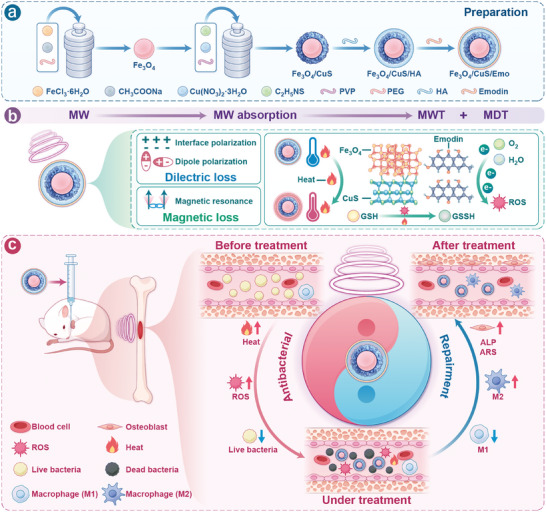
a) Schematic of Fe_3_O_4_/CuS/Emo preparation. b) The mechanism of microwave response of Fe_3_O_4_/CuS/Emo for the treatment of osteomyelitis: With the combined effect of heterojunction and emodin, the MA is enhanced, resulting in the synergistic effect of MWT and MDT. c) Mechanism of Fe_3_O_4_/CuS/Emo assisted microwave treatment of osteomyelitis: When Fe_3_O_4_/CuS/Emo nanoparticles enter the bone marrow cavity, they come into close contact with bacteria. Under the radiation of microwave, the enhanced MWT and MDT effects of Fe_3_O_4_/CuS/Emo nanoparticles will generate a lot of heat and ROS to killing bacteria. After the microwave, emodin released by Fe_3_O_4_/CuS/Emo nanoparticles can promote the transformation of macrophages from M1 type to M2 type, and also promote the differentiation and maturation of osteoblasts, thus contributing to the repair of bone tissue.

Bacteria can produce and release significant quantities of hyaluronidase to break down hyaluronic acid (HA) present in animal or human tissues. This aids in their spread and invasion during infections.^[^ ^19^ ^]^ To minimize the impact of microwaves on healthy tissues and improve the targeting ability of the heterojunction system, HA is deemed an optimal material to link the Fe_3_O_4_/CuS heterojunction with emodin. Based on the above ideas, we developed a multifunctional hybrid heterojunction system, Fe_3_O_4_/CuS/Emo nanoparticles, which offered a comprehensive “ one stone, two birds” capability. This included antibacterial and anti‐inflammatory effects. In the system, Fe_3_O_4_/CuS heterojunction was a fundamental nanoplatform with MWT and MDT integration capabilities that increase MA via heterogeneous surfaces. Emodin was immobilized on the surface of the heterojunction by HA as a cross‐linker (**Scheme** **1**a). After Fe_3_O_4_/CuS/Emo nanoparticles accumulated at the site of bacterial infection, the hyaluronidase secreted by the bacteria induces the enzymatic degradation of the HA in the nanoparticles,^[^ ^20^ ^]^ subsequently revealing the Fe_3_O_4_/CuS heterojunction. Under microwave radiation, the heterojunction formed in the Fe_3_O_4_/CuS system significantly amplified the interfacial polarization effect. The charge accumulation at this interface resulted in dipolar polarization, facilitating the efficient conversion of electromagnetic energy into thermal energy.^[^ ^21^ ^]^With the addition of emodin, its *π*‐conjugated structure accelerated the charge transfer between heterojunctions and released a large number of free electrons trapped by oxygen and polar molecules, thus increasing the production of ROS (Scheme 1b). By harnessing ample heat and ROS, this approach achieved remarkable antibacterial effects both in vitro and in vivo. Moreover, the unique anti‐inflammatory and antioxidant properties of emodin could aid in remodeling the immune microenvironment to promote bone repair at a later stage (Scheme 1c). Therefore, this treatment method will be a promising strategy for the treatment of osteomyelitis.

## 2 Results and Discussion

### 2.1 Preparation and Characterization of Fe_3_O_4_/CuS and Fe_3_O_4_/CuS/Emo

**Figure 1:**
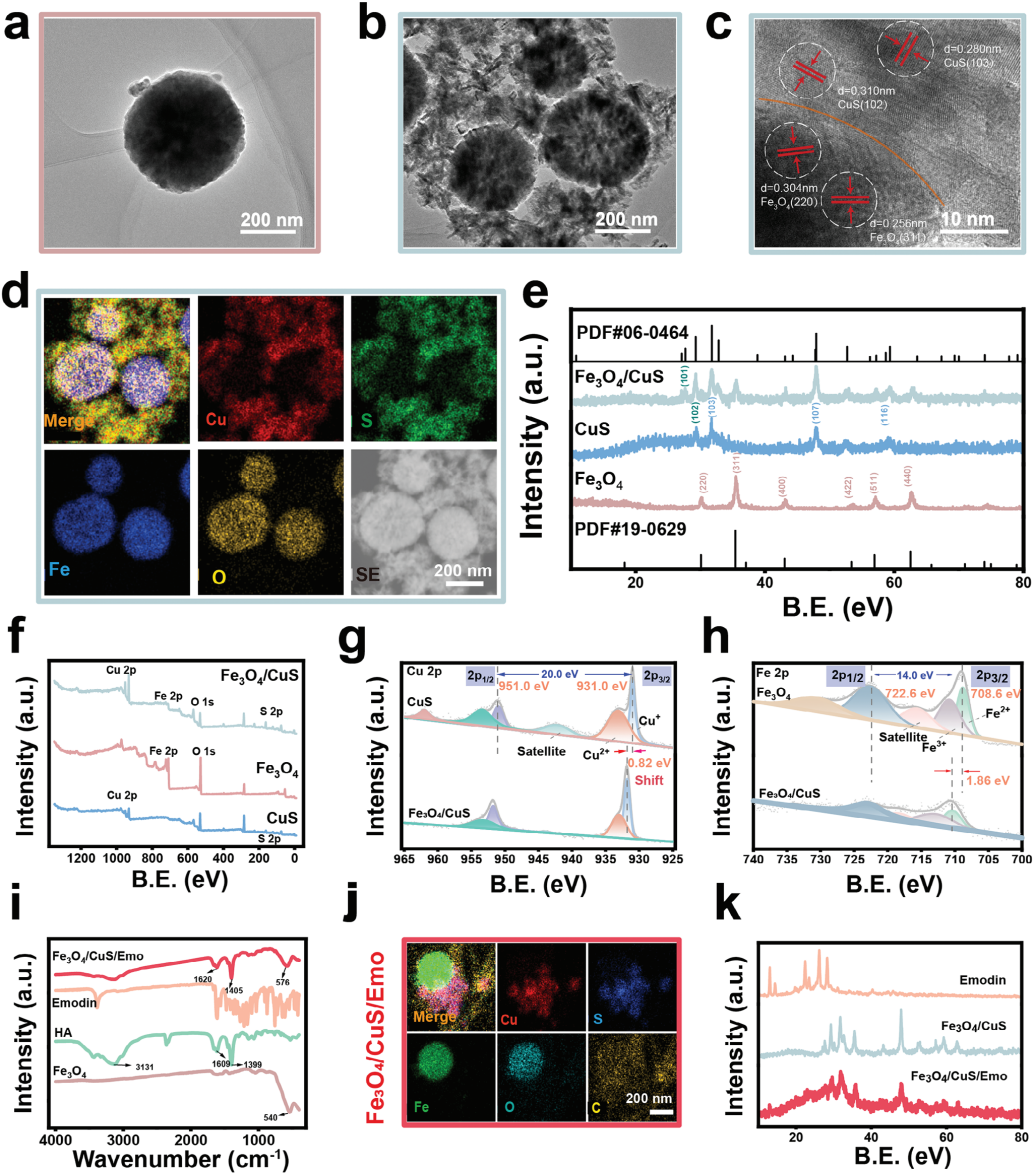
Basic characterization of Fe_3_O_4_/CuS and Fe_3_O_4_/CuS/Emo. a) TEM images of Fe_3_O_4_; b) TEM images of Fe_3_O_4_/CuS; (c) HRTEM images of Fe_3_O_4_/CuS, orange dashed lines represent distinct interfaces; d) EDS mapping of Fe_3_O_4_/CuS; e) XRD images of CuS, Fe_3_O_4_ and standard PDF cards; f) Full XPS spectrums of CuS, Fe_3_O_4_, Fe_3_O_4_/CuS; g) High‐resolution XPS maps of Cu from CuS and Fe_3_O_4_/CuS; h) High‐resolution XPS maps of Fe from Fe_3_O_4_ and Fe_3_O_4_/CuS. i) FTIR curves of Fe_3_O_4_, emodin, HA, and Fe_3_O_4/_CuS/Emo; j) EDS mapping of Fe_3_O_4/_CuS/Emo; k) XRD images of emodin, Fe_3_O_4/_CuS, and Fe_3_O_4/_CuS/Emo.

CuS and Fe_3_O_4_ were both synthesized by solvothermal methods.^[^ ^22^ ^]^ Initially, the independently synthesized CuS formed a flower‐like morphology with layered nanosheets, while Fe_3_O_4_ assumed a spherical morphology with a textured surface (Figure S1a,b, Supporting Information). Fe_3_O_4_/CuS was synthesized by two hydrothermal processes. Simply put, the Fe_3_O_4_ particles synthesized for the first time were added to the precursor solution of CuS to undergo a second hydrothermal process. As seen in Figure S2 (Supporting Information), CuS crystals had a large distribution on the Fe_3_O_4_ surface. The morphology of nanomaterials was further observed by TEM. TEM images showed that the initial Fe_3_O_4_ appeared spherical. After the hydrothermal process, it was surrounded by CuS forming a lamellar stack structure (**Figure** **1**a,b). High‐resolution transmission electron microscopy (HRTEM) images highlighted a distinct heterogeneous interface between CuS and Fe_3_O_4_, delineated by an orange dashed line in Figure 1c. The interplanar spacings of 0.31 and 0.28 nm could be ascribed to the (102) and (103) crystal planes of CuS, respectively.^[^ ^23^ ^]^ And interplanar spacing of 0.304 and 0.256 nm assigned to the (220) and (311) crystal planes of Fe_3_O_4_, respectively.^[^ ^24^ ^]^ The heterogeneous interfaces of the composite crystals could exhibit an accumulation and non‐uniform distribution of space charge, which induced polarization and relaxation effects, consequently attenuating electromagnetic waves.^[^ ^25^ ^]^ EDS mapping further showed a large distribution of CuS on Fe_3_O_4_, which confirmed that the two‐step hydrothermal method could realize the nucleation growth of CuS on Fe_3_O_4_ microspheres (Figure 1d). The X‐ray diffraction (XRD) pattern (Figure 1e) showed that Fe_3_O_4_/CuS had higher crystallinity than CuS and Fe_3_O_4_. Additionally, Fe_3_O_4_/CuS displayed a novel peak corresponding to the (101) facets of the CuS crystal, indicating that Fe_3_O_4_ doping potentially influenced the growth of CuS crystals. The full‐spectrum of XPS confirmed the presence of Cu, S, Fe, and O in Fe_3_O_4_/CuS (Figure 1f). High‐resolution XPS maps of Cu 2p and Fe 2p are shown in Figure 1g,h. In CuS crystals, the characteristic peaks at 951.0 and 931.0 eV of the binding energy corresponded to the Cu 2p_1/2_ and 2p_3/2_ orbitals, respectively.^[^ ^26^ ^]^ In Fe_3_O_4_ crystals, the characteristic peaks at 722.6 and 708.6 eV of the binding energy corresponded to the Fe 2p_1/2_ and 2p_3/2_ orbitals, respectively. The positive trivalent and positive bivalent of iron elements coexisted, which is consistent with the reports in the literature.^[^ ^27^ ^]^ Significantly, the Cu 2p_3/2_ and Fe 2p_3/2_ main peaks of the Fe_3_O_4_/CuS crystals exhibited a leftward shift, suggesting an elevation in binding energy. This shift likely resulted from the robust electronic interaction between CuS and Fe_3_O_4_, which further substantiated the formation of heterojunctions.^[^ ^28^ ^]^

To achieve the linkage of emodin, HA underwent intense shear forces, resulting in a polysaccharide coating with excellent mechanical adhesion on the surface of the Fe_3_O_4_/CuS heterojunction. During this process, the abundant carboxyl and hydroxyl groups in HA interacted with the metal ions present on the Fe_3_O_4_/CuS surface through electrostatic forces and coordination bonds.^[^ ^29^ ^]^ Additionally, the *π‐*conjugated structures and hydroxyl substituents in emodin could form hydrogen bonds with the carboxyl and hydroxyl groups of HA.^[^ ^30^ ^]^ Fourier transform infrared spectrum (FTIR) confirmed this phenomenon. As revealed in Figure 1i, the stretching vibration peak of Fe–O in Fe_3_O_4_ at 540 cm^−1^.^[^ ^31^ ^]^ Following the self‐assembly of HA with emodin, this peak shifted to 576 cm^−1^. Furthermore, in the Fe_3_O_4_/CuS/Emo group, absorption peaks at 1620 and 1405 cm^−1^ appeared, corresponding to the asymmetric stretching of the carboxyl group (C═O) and the symmetric stretching of C─O, respectively. This change arose from the alterations in the absorption peaks at 1609 and 1399 cm^−1^ attributed to the carboxyl groups of HA.^[^ ^32^ ^]^ From a micromorphology point of view, the SEM image of emodin revealed a feathery morphology (Figure S3, Supporting Information), and the morphology of heterojunction was similar before and after self‐assembly, as depicted in Figures 1j and S4 (Supporting Information). Differently, a thicker organic coating was formed around the Fe_3_O_4_/CuS heterojunction in the structure of Fe_3_O_4_/CuS/Emo. The anterior and posterior changes of emodin on Fe_3_O_4_/CuS heterojunction connections were also shown on the XRD test as well. As shown in Figure 1k, emodin exhibited peaks within the 10° to 30° range, which was also observed in the Fe_3_O_4_/CuS/Emo spectrum. The zeta potential test was used to measure the surface charge changes before and after the modification of nanoparticles. As shown in Figure S5 (Supporting Information), zeta potential measurements showed that both Fe_3_O_4_/CuS heterojunction and emodin exhibited negative potential. Due to the linking action of HA, the combined potential was further reduced to −20.56 ± 2.39 mV. This suggested that the stability of the composite particles was further strengthened in solution.

### 2.2 Enhanced MA Mechanism

**Figure 2:**
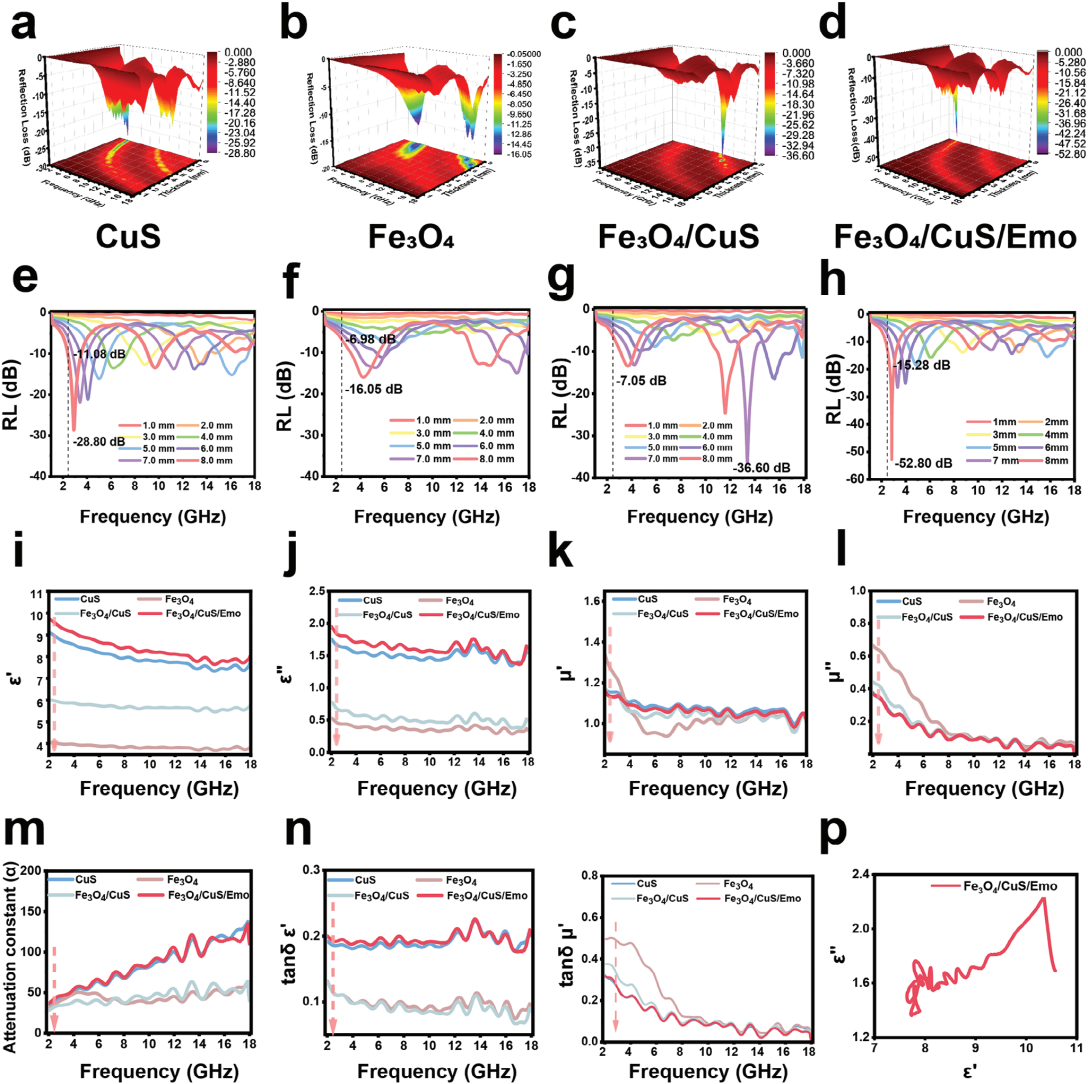
Enhanced MA mechanism. 3D frequency dependence graph: a) CuS; b) Fe_3_O_4_; c) Fe_3_O_4_/CuS; d) Fe_3_O_4_/CuS/Emo; Reflection loss curve: e) CuS; f) Fe_3_O_4_; g) Fe_3_O_4_/CuS; h) Fe_3_O_4_/CuS/Emo; i) The real part of the dielectric constant in the range of 2–18 GHz; j) The imaginary part of the dielectric constant in the range of 2–18 GHz; k) The real part of the permeability in the range of 2–18 GHz; l) The imaginary part of the permeability in the range of 2–18 GHz; m) The attenuation coefficient in the range of 2–18 GHz; n) The dielectric tangent curve in the range of 2–18 GHz; o) Magnetic tangent curve in the range 2–18 GHz; p) Cole–Cole curve of Fe_3_O_4_/CuS/Emo at 2–18 GHz.

To illuminate the MA mechanisms of the material, we characterized the reflection loss (RL) values along with the electromagnetic parameters of various samples across the 1–18 GHz frequency spectrum. The 3D RL mapping plots of CuS, Fe_3_O_4_, Fe_3_O_4_/CuS, and Fe_3_O_4_/CuS/Emo are shown in **Figure** **2**a–d. As depicted in Figure 2e–h, the corresponding 2D RL curves demonstrated the frequency and thickness dependence of the reflection loss characteristics. Specifically, the minimum RL values of CuS and Fe_3_O_4_, were −28.80 and −16.05 dB, respectively. Upon forming a heterojunction, the minimum RL value dropped to −36.6 dB. Remarkably, the introduction of emodin led to the Fe_3_O_4_/CuS/Emo achieving an impressive minimum RL of −52.80 dB, suggesting optimized MA ability. Furthermore, as the thickness increased, the minimum RL value of Fe_3_O_4_/CuS/Emo progressively shifted toward the lower medical frequency range. And the RL value reached −15.28 dB at the medical frequency (2.45 GHz). This trend could pave the way for the implementation of MW therapy.

The real and imaginary components of complex permittivity and complex permeability illustrate how well a material could store and dissipate electromagnetic waves.^[^ ^33^ ^]^ As shown in Figure 2i,j, the values of ε′ and ε′ of Fe_3_O_4_/CuS/Emo surpassed those of the other groups across the entire frequency range, indicating a greater capacity for dielectric storage and loss. In addition, the magnetic loss capability was also studied. As shown in Figure 2k,l, the *μ*′ and *μ*″ of Fe_3_O_4_/CuS/Emo were close to CuS at 2.45 GHz and smaller than Fe_3_O_4_/CuS and Fe_3_O_4_. The magnitudes of *ε*″ and *μ*″ played a decisive role in how effectively materials could absorb electromagnetic waves, ultimately influencing the attenuation coefficient (*α*) as a whole. The larger α value indicated that the electromagnetic wave decays more rapidly.^[^ ^34^ ^]^ As shown in Figure 2m, the *α* value of each group of samples rose with the increase in frequency, indicating a swift attenuation of microwaves at elevated frequencies. Among them, the value of *α* for Fe_3_O_4_/CuS/Emo was notably superior, suggesting a remarkable ability to dissipate microwaves. In addition, the dielectric loss tangent (tan *δ_ε_* = *ε*′/*ε*″) and magnetic loss tangent (tan *δ_μ_* = *μ*′/*μ*″) were employed to estimate the dielectric loss and magnetic loss capacities, respectively. Among the four groups of samples, the tan *δ_ε_* of Fe_3_O_4_/CuS/Emo was higher than that of the other groups at 2.45 GHz (Figure 2n,o). It could be summarized that the microwave loss of Fe_3_O_4_/CuS/Emo was primarily caused by dielectric loss. The dielectric losses dominated interface polarization and dipole polarization. They originated from heterogeneous interfaces and polar groups, such as ─OH and ─COOH from HA and emodin, respectively.

Subsequently, the Cole–Cole semicircular model was utilized to calculate the polarization relaxation process. Aligned with the Debye dipole relaxation theory, the semicircles in the Cole–Cole plot corresponded to the Debye relaxation processes, while the trailing tails indicated the conductivity.^[^ ^6c^ ^]^ The number of semicircles observed served as a measure of the intricacy involved in the polarization process. According to Figure 2p, the Fe_3_O_4_/CuS/Emo exhibited multiple polarization relaxation, which was related to interfacial polarization, dipole‐orientation polarization, and conductivity loss.^[^ ^35^ ^]^ In conclusion, the increase in dielectric loss of Fe_3_O_4_/CuS/Emo could be attributed to two points: first, the inhomogeneous heterogeneous interface in the bimetallic structure heightened the asymmetry of the space charge distribution. Second, a large number of polar groups acted as polarization centers, promoting dipole polarization.

### 2.3 Characterization of Fe_3_O_4_/CuS/Emo Enhanced MA Responsivity

**Figure 3:**
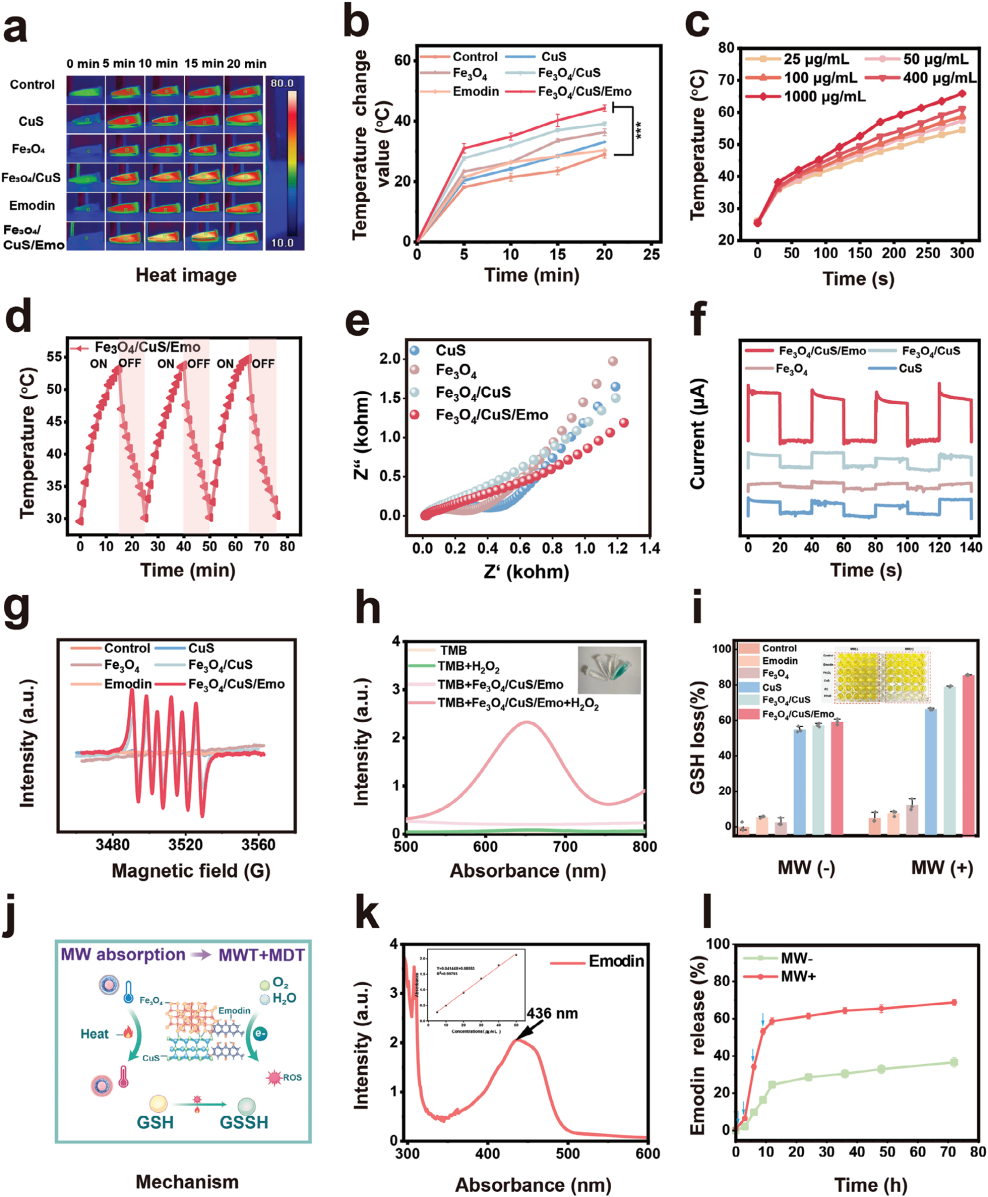
Characterization of Fe_3_O_4_/CuS/Emo MW responsivity. a) The heat images and b) the heating profiles of each group of samples (1 mg mL^−1^) were irradiated by MW at 2.45 GHz; c) Temperature rise curves of different concentrations within 5 min of irradiation at 10 W; d) Thermal stability of Fe_3_O_4_/CuS/Emo (25 µg mL^−1^) under MW irradiation at 5 W for 15 min; e) EIS curves of each group of samples under MW irradiation at 5 W; f) The MW response current curve of each group of samples; g) ESR spectra of Fe_3_O_4_/CuS/Emo and blank samples for ·O_2_^−^; h) UV–vis absorption peak of TMB; i) GSH loss rate in each group of samples (25 µg mL^−1^) after treatment with or without MW irradiation; j) Schematic diagram of GSH consumption under MW thermal and dynamic effects of Fe_3_O_4_/CuS/Emo; k) UV absorption curve and standard curve of emodin in ethanol; l) Emodin release curves of Fe_3_O_4_/CuS/Emo at specific time points without MW and after MW treatment.

The propagation of electromagnetic waves through a medium could lead to a loss of electromagnetic energy due to attenuation, with a significant portion being transformed into thermal energy.^[^ ^36^ ^]^ Thus, the in vitro heating capacity of each group could serve as a useful benchmark for assessing the effectiveness of MA.^[^ ^37^ ^]^ As demonstrated in **Figure** **3**a,b, the heating rate of Fe_3_O_4_/CuS/Emo considerably surpassed that of other groups. It illustrated the enhanced MW thermal effect of Fe_3_O_4_/CuS/Emo compared with other groups. In addition, Fe_3_O_4_/CuS/Emo exhibited concentration‐dependent with MW treatment (Figure 3c). Notably, after undergoing three heating and cooling cycles, there appeared to be no obvious change in the MW thermal response of Fe_3_O_4_/CuS/Emo (Figure 2c), which suggested a high level of thermal stability (Figure 3d).

Electrical resistance and current responses to MW irradiation were quantified using an electrochemical workstation. Figure 3e,f displayed the electrical impedance values in ascending order: Fe_3_O_4_/CuS/Emo < Fe_3_O_4_/CuS < CuS < Fe_3_O_4_. On the flip side, the current measurements during the three on–off cycles exhibit an inverse trend, signifying the most rapid charge transfer in the Fe_3_O_4_/CuS/Emo group when subjected to MW irradiation. The reason might be that there was a large amount of unevenly distributed charge at the heterogeneous interface, which tended to mobilize under microwave excitation. The *π*‐conjugated structure of emodin provided orbitals and additional charges that might significantly accelerate the motion of the charges. During the motion process, the charge could react with water and oxygen to form ROS. Consequently, based on the increased rate of charge transfer, we assumed that the heterogeneous interface and emodin could effectively enhance the MW catalytic performance. In order to measure the ROS production of Fe_3_O_4_/CuS/Emo under MW radiation, DMPO was used as the trapping agent of ·O_2_^−^. As shown in Figure 3g, Fe_3_O_4_/CuS/Emo produced more ROS (·O_2_^−^) under MW irradiation than other groups, suggesting that Fe_3_O_4_/CuS/Emo had a stronger MW catalytic ability compared to the other groups. This underpinned the potential of MDT to generate bursts of ROS.

Persistent bacterial infection produced a higher ROS content at the infection site (especially H_2_O_2_) than in normal tissues.^[^ ^38^ ^]^ 3,3′,5,5′‐Tetramethylbenzidine (TMB) was used to evaluate the capacity of Fe_3_O_4_/CuS/Emo to convert H_2_O_2_ into hydrogen peroxide (·OH). As shown in Figure 3h, colorless TMB was oxidized blue oxTMB in the presence of ·OH, which had a maximum absorption peak at 652 nm.^[^ ^39^ ^]^ In four groups, the TMB + Fe_3_O_4_/CuS/Emo + H_2_O_2_ group exhibited a significant absorption peak, while a negligible absorption peak was observed in other groups. This finding confirmed the ability of Fe_3_O_4_/CuS/Emo to catalyze the generation of a large number of ·OH in H_2_O_2_‐rich environments, thereby improving antimicrobial effectiveness. Glutathione (GSH) played a crucial role in safeguarding against internal oxidative stress in the bacteria. It was subsequently oxidized to glutathione disulfide (GSSG) when met ROS. This process could attenuate the therapeutic efficacy of MDT for antimicrobial action.^[^ ^40^ ^]^ Therefore, the ability of materials consuming GSH was investigated under MW action. As shown in Figure 3i, after MW irradiation, the colors of all the groups noticeably faded, approaching a nearly colorless state for Fe_3_O_4_/CuS/Emo. Their loss rates were calculated to be 5.02%, 7.55%, 12.30%, 66.31%, 79.06%, and 86.40%. This suggested that ROS produced by Fe_3_O_4_/CuS/Emo under MW irradiation could substantially diminish the levels of GSH. Because MW energy alone might not be sufficient to induce ROS production in water‐based media, we put forward a hypothesis of Fe_3_O_4_/CuS/Emo enhancing MW thermal and MW dynamic effects: the interplay between metal heterojunction and emodin‐endowed Fe_3_O_4_/CuS/Emo with enhanced MA and catalytic capacity. Consequently, Fe_3_O_4_/CuS/Emo could rapidly absorb microwaves to generate heat and accelerate charge transfer in polar media. As a result, during the charge transfer process, charges could react with the surrounding oxygen and water molecules to produce a large amount of ROS and deplete GSH.^[^ ^41^ ^]^

The emodin release profile of Fe_3_O_4_/CuS/Emo was investigated following MW treatment. Initially, the loading rate and individual mass content of emodin in Fe_3_O_4_/ CuS/Emo nanoparticles were determined to be 30.70 ± 0.53% and 5.09 ± 0.16% respectively, as established by the standard curve of emodin in ethanol (Figure 3k). The Fe_3_O_4_/CuS/Emo solution was divided into two groups: one without MW treatment and another subjected to intermittent MW exposure for 20 min across four times. As illustrated in Figure 3l, the MW‐treated group exhibited a significant emodin release of over 69.42% within 72 h, markedly surpassing the 37.27% release observed in the untreated group. The above results indicated that MW excitation accelerated the release of emodin from Fe_3_O_4_/CuS/Emo. It could provide a good basis for the subsequent exertion of anti‐inflammatory effects.

### 2.4 In Vitro Antibacterial Activity of Fe_3_O_4_/CuS/Emo

**Figure 4:**
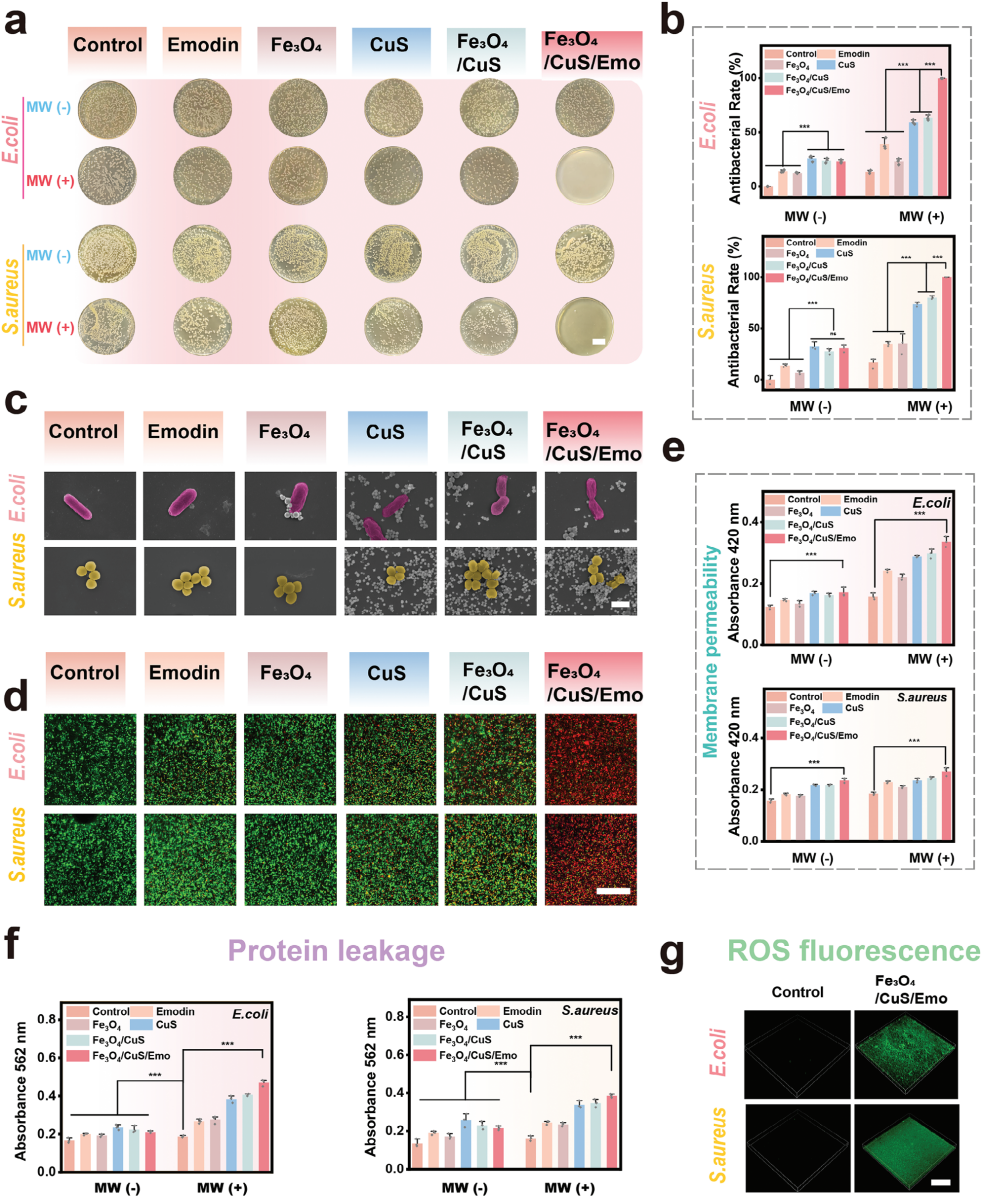
Evaluation of antibacterial performance and investigation of antibacterial mechanism in vitro. a) The coated images of *S. aureus* and *E. coli* of different samples; (Scale bar: 2 mm) b) The antibacterial rate of *S. aureus* and *E. coli* in each group; c) Scanning electron microscope images of *S. aureus* and *E. coli* in each group after MW treatment (scale bar: 1 µm); d) Live and dead staining images of *S. aureus* and *E. coli* in each group of samples after MW treatment (scale bar: 100 µm); e) Endometrial permeability of *S. aureus* and *E. coli* in each group; f) Protein leakage of *S. aureus* and *E. coli;* g) DFCH‐DA fluorescence images of *S. aureus* and *E. coli* in blank group and Fe_3_O_4_/CuS/Emo group after MW treatment (scale bar: 100 µm). *p < 0.05, **p < 0.01, ***p < 0.001, *n* = 3.

The plate coating method was used to assess antibacterial activity against *Escherichia coli* (*E. coli*) and *Staphylococcus aurus* (*S. aureus*). The efficacy of Fe_3_O_4_/CuS/Emo at varying concentrations was presented in Figure S6a,b (Supporting Information). A dose‐dependent enhancement in antimicrobial activity was noted. Notably, the concentration of 25 µg mL^−1^ yielded an antimicrobial rate ≈99% when MW treatment for both *E. coli* and *S. aureus*. Consequently, 25 µg mL^−1^ was chosen for further experimentation. As shown in **Figure** **4**a, all groups without MW treatment exhibited negligible bactericidal effects. Nevertheless, a pronounced decrease in bacterial colonies was observed across all groups when MW treatment, especially for Fe_3_O_4_/CuS/Emo group. Its efficacy in eliminating *E. coli* and *S. aureus* reached impressive rates of 99.99 ± 0.15% and 99.85 ± 0.12%, respectively (Figure 4b).

The morphology of bacteria after the MW treatment was observed by SEM. As shown in Figure 4c, in the Fe_3_O_4_/CuS/Emo group, bacteria showed more serious morphological collapse and damage compared to other groups. This was primarily due to the increased proximity of HA to bacteria and the synergistic effect of MWT and MDT.^[^ ^42^ ^]^ In addition, fluorescent staining was employed to visualize the distribution of viable and dead bacteria. As shown in Figure 4d, the nearly entirely stained red in the Fe_3_O_4_/CuS/Emo group meant excellent antibacterial activity with MW treatment. To further explore the effects of Fe_3_O_4_/CuS/Emo group with MW treatment on bacterial, the membrane permeability was examined. As shown in Figure 4e, the absorbance of the Fe_3_O_4_/CuS/Emo group increased more significantly when MW treatment than the other groups. This enhancement might be attributed to the improved thermal effect of MW on Fe_3_O_4_/CuS/Emo. Alterations in the permeability of bacterial membranes could lead to the release of internal substances, which prompted the use of the BCA protein kit to identify any leakage of bacterial proteins. As illustrated in Figure 4f, the Fe_3_O_4_/CuS/Emo group demonstrated a markedly greater absorbance during microwave treatment compared to the other groups, indicating a substantial release of bacterial proteins. DCFH‐DA was utilized to investigate ROS within bacteria. As illustrated in Figure 4g, a strong fluorescence signal was observed in the Fe_3_O_4_/CuS/Emo group, indicating that a significant amount of ROS was generated and infiltrated the bacteria. Furthermore, since bacterial internal GSH could offer protection against oxidative stress caused by external ROS, its levels were also assessed. There was a severe depletion of GSH in the Fe_3_O_4_/CuS/Emo group than the control group (Figure S7a,b, Supporting Information), which indicated an imbalance of redox reactions inside the bacteria. Thus, we believed that the excellent antibacterial activity of Fe_3_O_4_/CuS/Emo was attributed to the effect on the bacterial membrane and the generation of oxidative stress.

### 2.5 In Vitro Anti‐Biofilm Evaluation

**Figure 5:**
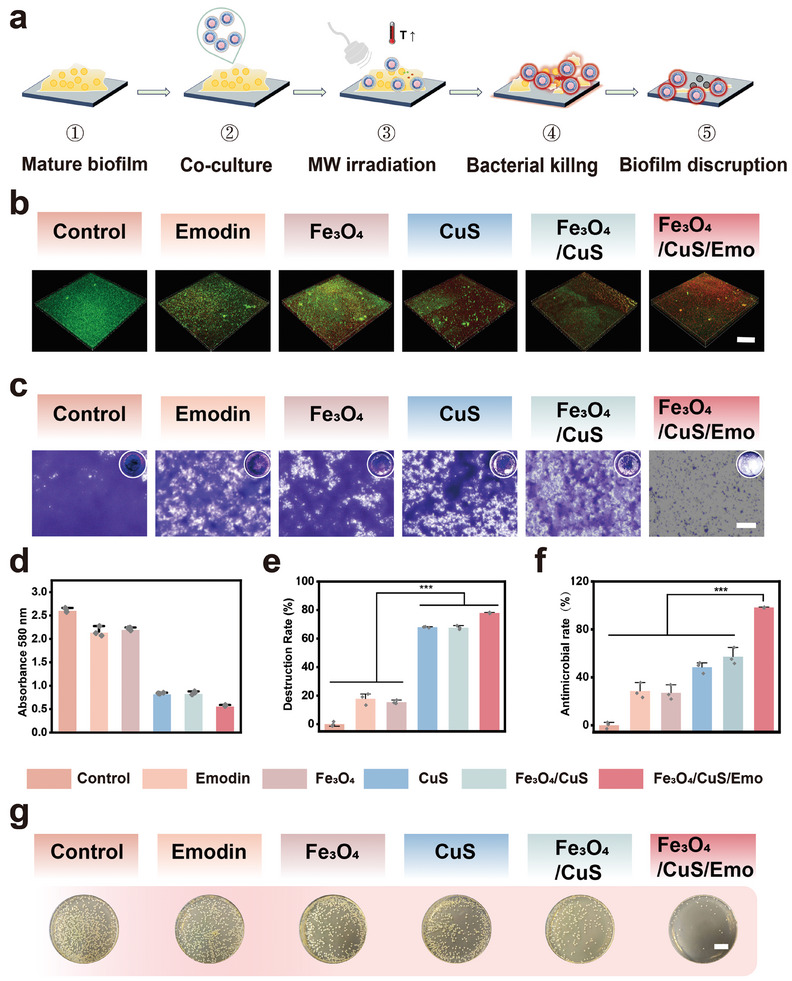
In vitro anti‐biofilm evaluation a) The schematic of the biofilm removal process of Fe_3_O_4_/CuS/Emo with MW treatment; b) Biofilm fluorescence images with MW treatment (Scale bar: 200 µm); c) Light microscopy images of crystalline violet staining with MW treatment. (Scale bar: 100 µm); d) Absorbance of crystal violet solution of biofilm; e) Biofilm destruction rate of each group of samples with MW treatment; f) Antimicrobial rate of biofilm coated plate statistics. g) The coated plate images of biofilm, (Scale bar: 2 mm) *p < 0.05, **p < 0.01, ***p < 0.001, *n* = 3.

The development of *S. aureus* biofilms contributed significantly to the intractability of chronic osteomyelitis. They served as both metabolic and physical shields for the bacteria, impeding the penetration of antimicrobial agents.^[^ ^43^ ^]^ Given that bacteria in biofilms were capable of synthesizing hyaluronidase in large quantities, which had a unique closeness to HA.^[^ ^42^ ^]^ Therefore, the ability of Fe_3_O_4_/CuS/Emo nanoparticles was investigated to penetrate and destroy biofilms in a short period of time. According to the steps in **Figure** **5**a, we first cultured the mature biofilms, then cocultured them with the material for 2 h, followed by MW irradiation. The confocal images of the biofilms are depicted in Figure 5b. A large number of nanoparticles could be observed distributed in the biofilm in the Fe_3_O_4_/CuS/Emo group, and the red fluorescence of biofilms was significantly higher than that of other groups. This indicated that Fe_3_O_4_/CuS/Emo could enter into the biofilm and cause a large degree of bacterial killing under microwave radiation. Crystal violet‐stained images of the biofilms were presented in Figure 5c, where the biofilm structure of the Fe_3_O_4_/CuS/Emo group appeared markedly disrupted. A quantitative assessment of crystal violet absorbance was conducted to gauge the effectiveness of anti‐biofilm activity, as illustrated in Figure 5d. The findings revealed that the Fe_3_O_4_/CuS/Emo group achieved a biofilm destruction rate of ≈77.94 ± 0.89%, markedly surpassing the rates observed in the other groups, as shown in Figure 5e. To evaluate the impact of MW treatment on bacterial viability within biofilms, the treated biofilms were collected, and performed agar plating. As revealed in Figure 5f,g, Fe_3_O_4_/CuS/Emo group exhibited a markedly reduced colony count, achieving an antibacterial rate of 95.82 ± 1.06%. In summary, the application of Fe_3_O_4_/CuS/Emo with microwave assistance has been proved effective in disrupting biofilm structures and eliminating internal bacteria. This progress was vital for the effective radical management of bacterial infections in chronic osteomyelitis.

### 2.6 In Vitro Cytotoxicity Assays

**Figure 6:**
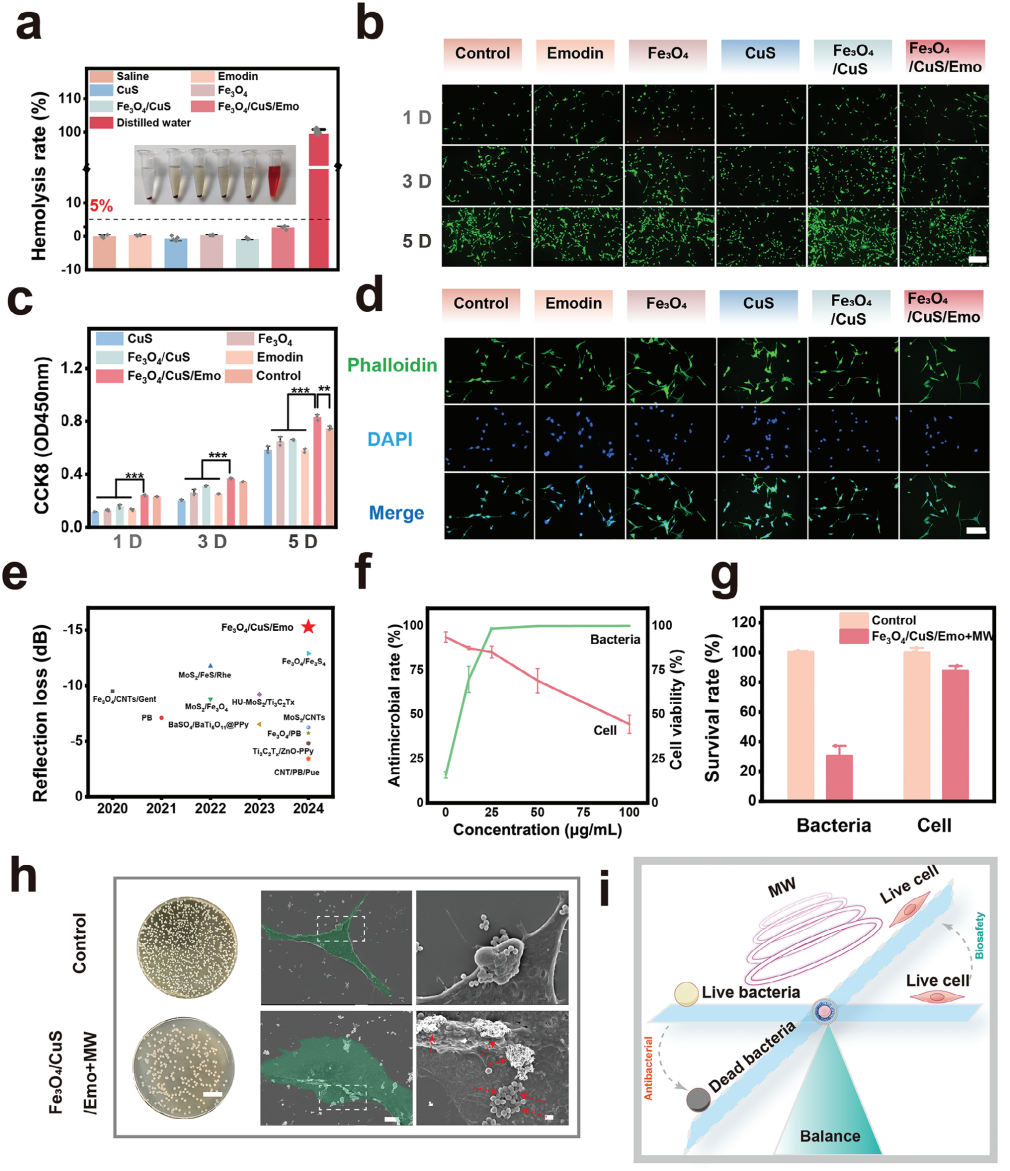
a) Hemolysis images and hemolysis rate statistics of materials in each group; b) Live and dead staining images of MC3T3‐E1 at day 1, 3, and 5 of each group of materials (scale bar: 100 µm); c) CCK8 solution OD values of MC3T3‐E1 at day 1, 3, and 5 of each group; d) The cytoskeletal morphology of MC3T3‐E1 at day 3, with actin staining in green and nuclear staining in blue (scale bar: 25 µm), *p < 0.05, **p < 0.01, ***p < 0.001, *n *= 3; e) Comparison of microwave absorption capacity of Fe_3_O_4_/CuS/Emo with published materials of the same type; f) Antimicrobial rate and cell viability of Fe_3_O_4_/CuS/Emo nanoparticle solutions with different concentrations under microwave irradiation; g) Bacterial and cell survival rates of Fe_3_O_4_/CuS/Emo nanoparticle solution (25 µg mL^−1^) combined with microwave radiation in the state of bacterial and cell coexistence; h) Images of bacterial coating plate of solution after microwave treatment (scale bar:2 mm) and SEM images of bacteria and cells after microwave treatment (scale bar:10 and 1 µm); i) Schematic diagram of the balance between bacteria and cells in microwave therapy.

The basic prerequisite for biomaterials in applications was their safety for biological systems.^[^ ^37^ ^]^ To evaluate blood safety, the hemolysis rate of the materials was analyzed. As illustrated in **Figure** **6**a, there was no considerable hemolysis (under 5%) of red blood cells in any of the groups, demonstrating their remarkable compatibility with blood. To further evaluate the long‐term biocompatibility profiles, original rat osteoblast precursor cells (MC3T3‐E1) were treated with materials. The cells were stained on days 1, 3, and 5 to ascertain the viability distribution (Figure 6b). The live‐dead staining results indicated a predominance of green fluorescence across all sample groups, signifying high cell survival rates. Additionally, the CCK8 assay was employed to quantitatively evaluate cell proliferation. As demonstrated in Figure 6c, cell proliferation was evident in all sample groups over time. However, when compared to the control group, all groups except for the Fe_3_O_4_/CuS/Emo group exhibited a trend toward reduced growth rates. Skeletal staining images of MC3T3‐E1 cells were revealed in Figure 6d. The cells across all groups displayed well‐defined cytoskeletons and extended pseudopodia. Based on these observations, we hypothesized that the enhanced cell proliferation observed in the Fe_3_O_4_/CuS/Emo group might be attributed to the release of low concentrations of ions (such as copper ions and iron ions) and emodin, which could promote the proliferation of osteoblasts.^[^ ^44^ ^]^ In conclusion, Fe_3_O_4_/CuS/Emo demonstrated outstanding biocompatibility and held significant promise for in vivo therapeutic applications.

### 2.7 The Balance between Antibacterial and Cytocompatibility

In this study, the excellent antibacterial properties (Section 2.5.) and good biocompatibility (Section 2.6) of the Fe_3_O_4_/CuS/Emo have been verified. However, it was a key issue to balance the relationship between the two. Comparison with published materials of the same type, Figure 6e showed that Fe_3_O_4_/CuS/Emo had more outstanding microwave absorption performance.^[^ ^6^, ^21^, ^45^ ^]^ This meant it could achieve antibacterial effects with lower material concentration and smaller microwave power, reducing damage to cells. Under mild microwave radiation, comparing cell viability and antibacterial rate of Fe_3_O_4_/CuS/Emo with different concentrations provided an important way to find the balance between antibacterial and cell safety. Encouragingly, from the double line graph including cell viability and antibacterial rate, we could find that the intersection was near 25 µg mL^−1^. At this concentration, cell viability was 85.18 ± 3.34%, and the antibacterial rate was as high as 98.50 ± 0.36% (Figure 6f). This indicated that this concentration might be the balance point between antibacterial and safety. To further verify the ability of Fe_3_O_4_/CuS/Emo to balance antibacterial properties and cell compatibility, we designed an experiment to treat the coexistence of bacteria and cells under mild power radiation. The cell viability determined by CCK8 is shown in Figure 6g. The survival rate of the treatment group was 87.8 ± 2.33%, still maintaining high physiological activity. Then, the co‐cultured solution was plated and counted. The plating image is shown in Figure 6h. It could be seen that the number of bacteria under the combined action of Fe_3_O_4_/CuS/Emo and microwave radiation was significantly reduced. Compared with the blank group, the bacterial survival rate dropped to 30.39 ± 6.15%. In addition, the morphology of irradiated bacteria and cells was observed by SEM, as shown in Figure 6h. In the same field of view, most of the bacteria in close contact with the material have collapsed ruptured cell membranes and even cytoplasmic leakage. While the cell membrane of the cell was intact without obvious damage. The reason for this difference might be the huge physiological structural differences between cells and bacteria. Cells were more complex in structure than bacteria. The cell membrane of cells contained a variety of complex lipid and protein components, which might have stronger resistance to thermal effects and ROS.^[^ ^46^ ^]^ In addition, when interacting with materials and microwave radiation, the abundant organelles and antioxidant enzymes of the cell might participate in the defense and repair mechanisms of the cell.^[^ ^47^ ^]^ In conclusion, at an appropriate concentration and microwave power, this therapy could balance antibacterial performance and cell compatibility well (Figure 6i). This advantage would expand a broader space for its application in the antibacterial field and was expected to minimize the adverse effects on cells while ensuring the antibacterial effect.

### 2.8 Immunomodulatory and Bone Promoting Effects of Fe_3_O_4_/CuS/Emo

**Figure 7:**
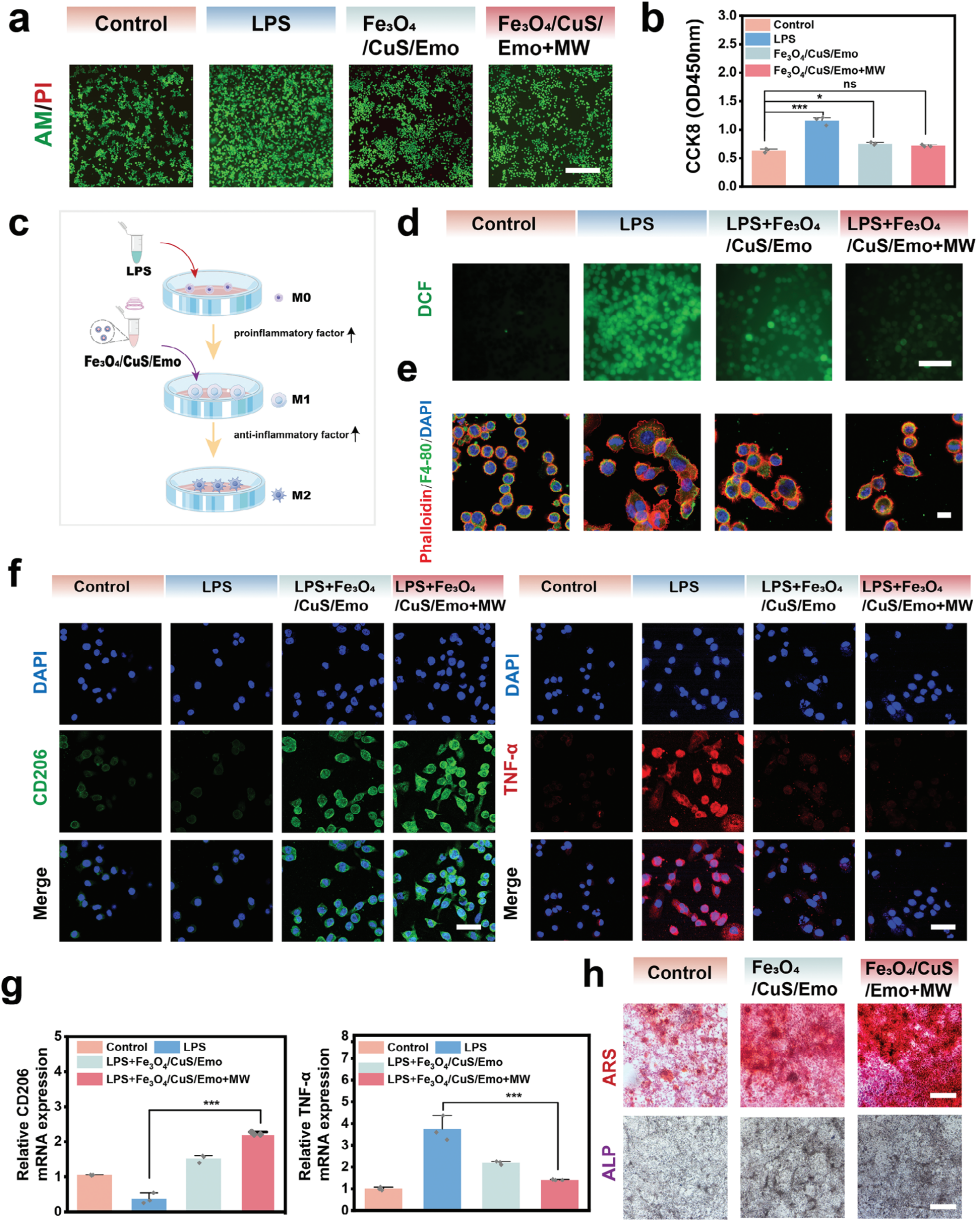
a) Live‐dead staining of RAW 264.7 for each group at day 1. (Scale bar: 50 µm); b) The OD values of CCK8 solution at day 1; c) Schematic diagram illustration of the experimental flow of Fe_3_O_4_/CuS/Emo used to reduce inflammation; d) DFCH‐DA staining fluorescence images of RAW 264.7 oxidative stress (Scale bar: 25 µm); e) Skeletal staining of macrophages with actin staining in red, F4/80 protein staining in green and nuclear staining in blue. (scale bar: 20 µm); f) Immunofluorescence staining of RAW 264.7, M2: CD206, M1: TNF‐α (Scale bar: 200 µm); g) Relative gene expression levels of anti‐inflammatory group CD206 and pro‐inflammatory gene TNF‐α at 14 days of culture, *p < 0.05, **p < 0.01, ***p < 0.001, *n *= 3; h) ALP staining and ARS staining of each group at 14 days of incubation (Scale bar: 100 µm).

After removing bacteria, the osteomyelitis lesion remained in an inflammatory stress state, which made it challenging for the bone to reconstruct during the repair process.^[^ ^48^ ^]^ Previous research indicated that pro‐inflammatory processes facilitated bacteria clearance from infected wounds, while an anti‐inflammatory macrophage‐enriched environment promoted tissue and wound regeneration.^[^ ^48^, ^49^ ^]^ Fe_3_O_4_/CuS/Emo was previously demonstrated to have excellent antibacterial ability, so we next investigated the effect of Fe_3_O_4_/CuS/Emo on macrophage remodeling in the repair process. First, the cytocompatibility of Fe_3_O_4_/CuS/Emo was further studied by live/dead staining and CCK8 assays (**Figure** **7**a,b). Based on the oxidative stress‐relieving and anti‐inflammatory effects of emodin, we explored the immunoregulatory capacity of Fe_3_O_4_/CuS/Emo on macrophages (Figure 7c). RAW 264.7 cells were stimulated with Lipopolysaccharides (LPS) to induce a pro‐inflammatory M1 phenotype. However, after treated with Fe_3_O_4_/CuS/Emo or Fe_3_O_4_/CuS/Emo + MW, the expression of ROS decreased more than in the LPS group (Figure 7d). It indicated that oxidative stress inside macrophages was alleviated. In addition, the long‐term effects of Fe_3_O_4_/CuS/Emo with MW treatment on macrophages were explored. Cytoskeletal staining (Figure 7e) revealed distinct morphologies: the majority of cells in the control group were spherical, those in the LPS group exhibited a spread appearance, and cells co‐cultured in the LPS + Fe_3_O_4_/CuS/Emo group or LPS + Fe_3_O_4_/CuS/Emo + MW group showed a shuttle‐like shape. Immunofluorescence was used to further characterize the phenotype of macrophages, which were stained for M2 markers (CD206) and M1 markers (TNF‐α), respectively.^[^ ^50^ ^]^ As shown in Figure 7f,a distinct red fluorescence of TNF‐α was detected in the LPS group. In contrast, the red fluorescence in LPS + Fe_3_O_4_/CuS/Emo or LPS + Fe_3_O_4_/CuS/Emo + MW group was attenuated. The green fluorescence of CD206 was enhanced in the above two groups. Furthermore, the findings from the RT‐PCR analysis indicated a rise in the expression level of the anti‐inflammatory gene, while the expression level of the pro‐inflammatory gene saw a decline (Figure 7g). The above results indicated that Fe_3_O_4_/CuS/Emo could modulate the inflammatory levels of macrophages in the late stage of the treatment. Surprisingly, Fe_3_O_4_/CuS/Emo showed stronger anti‐inflammatory effects after MW treatment. This was probably attributed to the accelerated release of emodin and HA by MW.^[^ ^51^ ^]^

Given that osteomyelitis typically led to significant bone destruction and loss, it was imperative that the treatment material should possess bone tissue repair capabilities.^[^ ^51a,b^ ^]^ The in vitro osteogenic ability of Fe_3_O_4_/CuS/Emo was assessed, and ALP and ARS were utilized as symbols for osteoblast differentiation. As depicted in Figure 7h, the ALP color in Fe_3_O_4_/CuS/Emo group and Fe_3_O_4_/CuS/Emo + MW group was darker than that of the control group after 14 days. Additionally, a greater number of calcium nodules and more pronounced red staining were observed in both Fe_3_O_4_/CuS/Emo and Fe_3_O_4_/CuS/Emo + MW groups. These results indicated that Fe_3_O_4_/CuS/Emo had the potential to mitigate oxidative stress in inflammatory cells, restructure the immune microenvironment, and enhance osteoblast differentiation. The osteogenic ability might contribute to the osteomyelitis treatment in the bone repair process.

### 2.9 In Vivo Treatment of Osteomyelitis

**Figure 8:**
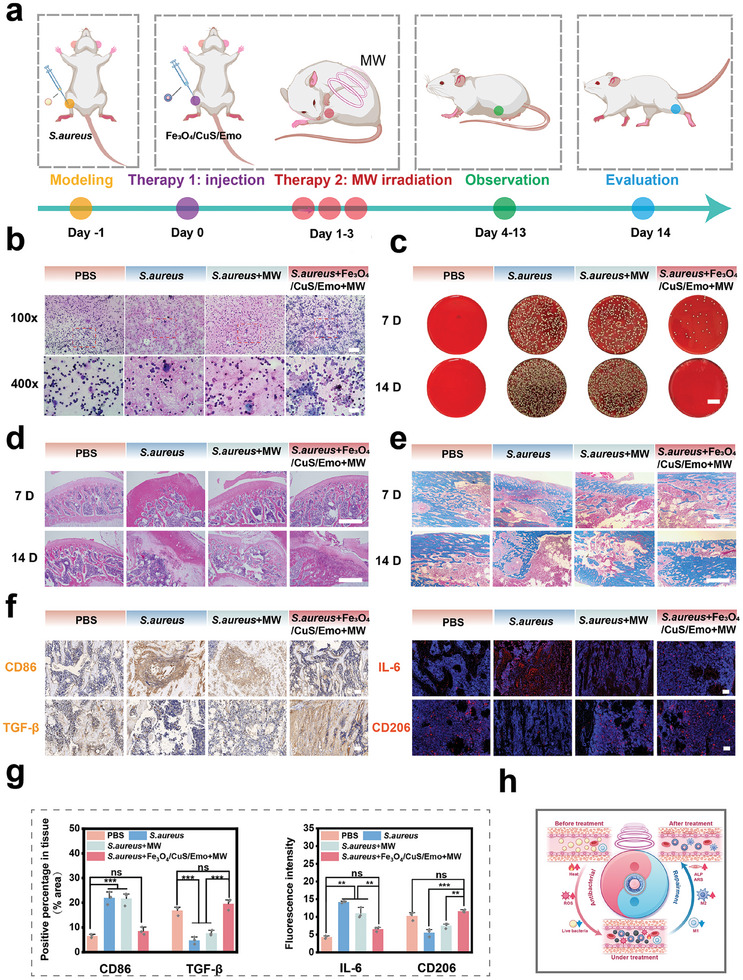
In vivo antibacterial activity and the treatment of osteomyelitis. a) Schematic diagram of the procedure for treating *S. aureus*‐infected osteomyelitis; b) Wright‐stained images of infected bone marrow tissues at day 7 (scale bars: 200 and 50 µm); c) Colony smear pictures of bone marrow at day 7 and 14 (scale bar:2 mm); d) HE staining of infected bone tissue at day 7 and 14 of treatment (scale bars:100 µm); e) Masson staining pictures of infected bone tissue at day 7 and 14 (scale bar: 50 µm); f) Immunohistochemical staining of CD86 and TGF‐β in infected tissue at day 14, M1:CD86, M2:TGF‐β (scale bar: 100 µm); Immunofluorescence staining of IL‐6 and CD206 at day 14, M1: IL‐6, M2: CD206 (scale bar: 100 µm); g) Statistical graph of the percentage of positive cells in CD86 and TGF‐β; Statistical graph of fluorescence intensity in CD206 and IL‐6. *p < 0.05, **p < 0.01, ***p < 0.001, *n* = 3; h) Diagram of Fe_3_O_4_/CuS/Emo microwave‐assisted technique for bone sterilization and tissue regeneration.

Considering the excellent in vitro antimicrobial properties and biocompatibility of Fe_3_O_4_/CuS/Emo, a model of osteomyelitis induced by *S. aureus* was designed to study further application. The modeling and treatment process are shown in **Figure** **8**a. After 7 days of treatment, right staining of bone marrow showed that a large number of immune cells appeared in each group (Figure 8b). To further illustrate the ability of Fe_3_O_4_/CuS/Emo with MW treatment to eliminate the bacterial burden in vivo, infected tibial bone marrow tissue was gathered at days 7 and 14 after treatment. Then the infected bone marrow tissue was cultured after dilution for bacterial counting. The plate coating images of bone marrow tissues (Figure 8c) revealed that MW treatment alone did not significantly reduce bacterial colonization. Conversely, after Fe_3_O_4_/CuS/Emo and MW treatment, very few bacteria were found at day 7, and no obvious bacteria were found at day 14. This was mainly because Fe_3_O_4_/CuS/Emo was able to exert MW thermal and dynamic effects to achieve in situ antibacterial activity in rats. Micro‐CT images taken two weeks after surgery were compared to assess bone infections in different groups. Figure S8 (Supporting Information) illustrated that the *S. aureus* group exhibited considerable bone damage and a tendency for spread at the surgical site, compared to the PBS group, with the *S. aureus *+ MW group showing less severity. Notably, in the *S. aureus *+ MW + Fe_3_O_4_/CuS/Emo group, the bone defect was significantly smaller. H&E staining of infected bone tissue was demonstrated in Figure 8d. On the 7th day, the bone tissue in the *S. aureus* group exhibited signs of redness, swelling, local uplift, and deformation. The *S. aureus *+ MW group displayed similar, albeit slightly less severe, symptoms. These changes were attributable to the infection caused by *S. aureus*, which led to local tissue congestion and edema, resulting in the observed alterations in bone tissue morphology. As the culture period progressed to the 14th day, both the *S. aureus* and *S. aureus *+ MW groups experienced trabecular fractures and a significant loss of bone integrity, indicating serious damage to the bone tissue. Furthermore, an increase in the number of inflammatory cells suggested that the inflammatory response was intensifying, likely due to the ineffective control of the infection, which facilitated the spread of inflammation and subsequent damage to the bone tissue. Although MW treatment demonstrated a mild alleviating effect on the inflammatory response at day 7, this effect was not significant by day 14, as it did not completely halt the progression of the infection. In contrast, the bone trabeculae in the PBS group and the *S. aureus *+ Fe_3_O_4_/CuS/Emo + MW group remained intact, with only minimal infiltration of inflammatory cells. In addition, Masson staining (Figure 8e) demonstrated that both the *S. aureus* group and the *S. aureus *+ MW group had severe bone tissue destruction over time due to bacterial infections. In contrast, in *S. aureus *+ Fe_3_O_4_/CuS/Emo + MW group, more mature collagen deposits and large amounts of new bone formation were observed. The above evidences confirmed that the treatment of Fe_3_O_4_/CuS/Emo under MW irradiation had effective antibacterial ability in vivo, which could effectively control bacterial infection and promote bone repair and regeneration. Additionally, HE staining of the heart, liver, spleen, lung, and kidney (Figure S9, Supporting Information) in each group did not reveal obvious lesions. This indicated that the therapeutic system was safe biologically.

Once the bacterial infection had been fully eradicated, it was essential to establish an ideal setting that fostered the healing and restoration of bone tissue. To gain further study the role of Fe_3_O_4_/CuS/Emo with MW treatment during osteomyelitis treatment, we conducted immunofluorescence staining and immunohistochemical tests on infected bone tissues from each group at day 14. As shown in Figure 8f,g, the M1 type macrophage markers (IL‐6 and CD86) were strongly expressed in the *S. aureus* and *S. aureus *+ MW groups due to severe bacterial infections. However, the expression was substantially reduced in the *S. aureus *+ Fe_3_O_4_/CuS/Emo + MW group due to the effective clearance of bacterial infection. In addition, the M2‐type macrophage markers (CD206 and TGF‐β) were expressed higher in the *S. aureus *+ Fe_3_O_4_/CuS/Emo + MW group than those in the above two groups. The results suggested that Fe_3_O_4_/CuS/Emo with MW treatment was beneficial to the formation of an anti‐inflammatory microenvironment after infection came under control and enhanced bone repair (Figure 8h).

## 3 Conclusion

In summary, this study has successfully developed a multifunctional hybrid heterojunction that achieves a “one stone, two birds” effect, effectively treating bacterial‐induced osteomyelitis under microwave stimulation. The combination of Fe_3_O_4_/CuS and emodin enhanced the microwave absorption and catalytic capabilities of the heterojunction system. The heat generated by the microwave energy loss increased the permeability of the bacterial cell membrane, facilitating the infiltration of catalytically generated ROS to penetrate into the bacterial interior and disrupt the redox balance. In synergy with MWT and MDT, Fe_3_O_4_/CuS/Emo demonstrated excellent antimicrobial capacity both in vitro and in vivo. Moreover, following an extended cultivation period, Fe_3_O_4_/CuS/Emo demonstrated a notable ability to eliminate surplus ROS, foster the shift of macrophages toward anti‐inflammatory states, and expedite the bone regeneration process, thereby enhancing recovery efforts in osteomyelitis therapy. This study presents a safe and efficient treatment of bacterial‐infected osteomyelitis by MW‐assisted therapy.

## 4 Experimental Section

### Chemicals and Materials Preparation

The chemicals and steps used in the material synthesis process are listed in the Supporting Information.

### Materials Characterization

The microstructure and the distribution of the corresponding elements were visualized by field emission scanning electron microscopy (FE‐SEM, JSM‐7001F, JEOL) and high‐resolution transmission electron microscopy (HR‐TEM, JEM‐2010, JEOL). XRD (Rigaku Dmax‐3C) was employed to acquire information about the crystal structure of the materials. A Cu target was the radiation and 2*θ* is from 10° to 80° in steps of 0.02°. The component chemical valence information of the elements on the surface of materials was detected by X‐ray photoelectron spectroscopy (XPS, Kα, Thermo). FTIR (Bruker Alpha, Germany) was utilized to observe alterations in various chemical bonds. The zeta potential and particle size of the particles were determined by nanoparticle sizer measurements (BeNano 180 Zeta Pro, Dandong, China).

### MA Parameter Measurement

One milligram of CuS, Fe_3_O_4,_ Fe_3_O_4_/CuS, and Fe_3_O_4_/CuS/Emo were respectively combined with a paraffin matrix to produce a coaxial ring. The electromagnetic properties are assessed within the 1–18 GHz frequency range using a vector network analyzer (Agilent Technologies E5071C). The MA performance of CuS, Fe_3_O_4,_ Fe_3_O_4_/CuS, and Fe_3_O_4_/CuS/Emo was detected by the reflection loss value (RL), calculated by Equations (1) and (2):

(1) $$Z_{\mathrm{in}}=\sqrt{\left|\mu_{r}/\varepsilon_{r}\right|}\;\tanh\left[j\left(\frac{2\pi df}{c}\right)\sqrt{\mu_{r}\varepsilon_{r}}\right]$$

(2) $$RL=20\left|\frac{Z_{\mathrm{in}}-1}{Z_{\mathrm{in}}+1}\right|$$

Among these variables, *Z*_in_ represents the normalized input impedance, while *μ*_r_ and *ε*_r_ stand for the relative permeability and dielectric constant. Furthermore, *f* denotes the frequency, *d* signifies the thickness of the absorber, and *c* represents the speed of light.

The attenuation constant (*α*) was calculated by Equation (3):

(3) $$\alpha =\frac{\sqrt{2}\pi f}{c}\sqrt{\left(\mu^{\prime \prime }\varepsilon^{\prime \prime }-\mu^{\prime }\varepsilon^{\prime }\right)+\sqrt{{\left(\mu^{\prime \prime }\varepsilon^{\prime \prime }-\mu^{\prime }\varepsilon^{\prime }\right)}^{2}+{\left(\mu^{\prime \prime }\varepsilon^{\prime }+\mu^{\prime }\varepsilon^{\prime \prime }\right)}^{2}}}$$

The dielectric loss (tan *δ*_E_) and magnetic loss (tan *δ*_M_) were calculated by Equations (4) and (5):

(4) $$\tan\delta_{\varepsilon }=\frac{\varepsilon^{\prime }}{\varepsilon^{\prime \prime }}$$

(5) $$\tan\delta_{\mu }=\frac{\mu^{\prime }}{\mu^{\prime \prime }}$$

In these variables, *μ*″ and *μ*′ denote the imaginary and real parts of the permeability, *ε*″ and *ε*′ represent the imaginary and real parts of the dielectric constant, respectively.

### MW Thermal Effect Measurements

With saline as a blank control, emodin, Fe_3_O_4_, CuS, Fe_3_O_4_/CuS, and Fe_3_O_4_/CuS/Emo (1 mg mL^−1^) solution were irradiated with MW at 10 W (2.45 GHz, Schneider Medical Equipment Co., Ltd., China) for 5 min. After 5 min, the power was decreased to 5 W to continue irradiation for 15 min. The instantaneous temperatures were documented with a near‐infrared thermometer gun (Hikmicro). And Fe_3_O_4_/CuS/Emo were irradiated at a power of 10 W, across varying concentrations (25, 50, 100, 400, and 1000 µg mL^−1^) for a duration of 5 min. In addition, to investigate the MW thermal stability of the Fe_3_O_4_/CuS/Emo (25 µg mL^−1^), three on/off cycles were performed under 5 W power.

### MW Catalysis Determination

The MW catalytic performance was primarily evaluated by measuring the generation of ROS. The electron paramagnetic resonance (EPR) spectra of the superoxide radical (·O_2_^−^) were obtained using the electron spin resonance spectrometer (EMXPLUS10/12, Bruker). The DMPO solution (methanol as solvent) was used to selectively capture the superoxide radical (·O_2_^−^). In the process, the sample (2 mg) was added to 1 mL of DMPO solution irradiated by MW. Subsequently, the mixture was transferred to EMxPLUS 10/12 for the identification of ROS.

### MW Response Current and Electrochemical Impedance Assessment

The MW electrochemical properties were measured by an electrochemical workstation (CHI660E, Shanghai, China). The reference electrode utilized was Ag/AgCl, with the counter electrode being Pt, and the working electrode being the experimental sample. Furthermore, the experimental samples were created by dispensing 200 µL of solution (2 mg mL^−1^) onto a 1 cm × 1 cm conductive ITO glass and air‐dried. And the electrolyte was 0.1 m Na_2_SO_4_ (99%, Aladdin, Shanghai) solution. Moreover, MW was used as an external energy source during the measurement of electrochemical impedance and MW response current.

### TMB Colorimetric Reaction Experiment

The ─OH production was determined by the colorimetric reaction of TMB. TMB solution (2 mm) was mixed with acetate buffer, Fe_3_O_4_/CuS/Emo solution, and Fe_3_O_4_/CuS/Emo with H_2_O_2_ solution, respectively. Then they were placed for 5 min away from light. UV spectrophotometer (UV‐1800, SHIMADZU) was used to measure the absorbance curve.

### GSH Consumption Experiment

With saline as a blank control group, emodin, CuS, Fe_3_O_4_, Fe_3_O_4_/CuS, and Fe_3_O_4_/CuS/Emo (25 µg mL^−1^, 1.7 mL) solutions were added to GSH carbonate buffer solution (1 mm, 0.3 mL). The conditions were divided into MW irradiation and non‐irradiation for 20 min. After treatment, DNTB (3.0 mg mL^−1^, 0.1 mL) was added and left for 30 min. The absorbance at 412 nm was determined using a UV spectrophotometer. The GSH consumption is calculated using the Equation (6).

(6) $$\mathrm{GSH}\;\;\mathrm{loss}=\frac{\left(\mathrm{Ab}s_{\mathrm{control}}-\mathrm{Ab}s_{\mathrm{sample}}\right)}{\mathrm{Ab}s_{\mathrm{control}}}\times 100\%$$

where *Abs*_control_ is the absorbance of the blank group, and *Abs*_sample_ is the absorbance of each experimental group.

### The Release Curve of Emodin

Determination of standard curve: the absorbance values at 436 nm were determined for different concentrations of emodin in ethanol solution (10, 20, 30, 40, and 50 µg mL^−1^), respectively. The standard curve of emodin in ethanol solution was derived. A mixture containing Fe_3_O_4_/CuS/Emo (5 mg) and PBS solution (4 mL) was placed into a dialysis bag (MWCO 3500 Da). The bag was then submerged in a PBS buffer with gentle agitation at 100 rpm and a temperature of 37 °C. MW irradiation was performed for 20 min at predetermined times (0, 4, 8, and 12 h). Solution (2 mL) was collected at a specific time. The collected solutions were freeze‐dried and dissolved with an ethanol solution. The absorbance value at 436 nm was correlated to the standard curve to determine the exact concentration.

### In Vitro Antimicrobial Tests

The antibacterial activity in vitro was assessed via the plate coating method. First, the bacteria solution was treated with saline to ≈2 × 10^7^ CFU mL^−1^. As saline as the blank control group, the diluted bacterial suspensions were incubated with saline, emodin, Fe_3_O_4_, CuS, Fe_3_O_4_, and Fe_3_O_4_/CuS/Emo at 25 µg mL^−1^ concentrations. The experimental conditions were divided into MW treatment and non‐MW treatment. Then the MW groups were irradiated with 10 W power for 5 min to rapidly heat up to 50 °C. After that, the temperature was maintained at ≈50 °C for 15 min. Finally, the bacterial solution was diluted to 1 × 10^3^ CFU mL^−1^, and 100 µL was evenly distributed on the agar plate and incubated at 37 °C for 24 h. The antibacterial rate was calculated according to Equation (7):

(7) $$\mathrm{Antibacterial}\;\mathrm{efficiency}=\frac{\left(N_{\mathrm{control}}-\;N_{\mathrm{sample}}\right)}{N_{\mathrm{control}}}\times 100\%$$

where *N*_control_ is the average number of colonies in the blank control group and *N*_sample_ is the average number of colonies in each experimental group.

To investigate further the antimicrobial properties of the samples, live/dead fluorescent staining of the bacteria was applied. The morphology of bacteria after MW treatment was examined by SEM. Then ONPG was utilized to determine the bacterial intimal permeability and the BCA protein test kit was applied to assess the extent of protein leakage. Finally, the DFCH‐DA probe was employed to determine ROS levels within bacteria, and DTNB was used to detect GSH loss in bacteria, according to the literature.^[^ ^52^ ^]^

### In Vitro Antibiofilm Properties

The antibiofilm activity in vitro was assessed via crystal violet staining. First, 1 mL *S. aureus* (≈1 × 10^8^ CFU mL^−1^) was placed in a 24‐well plate and incubated at 37 °C for 48 h. The material solution was added and co‐cultured at 37 °C for 2 h and irradiated with microwave for 20 min. After adding 200 µL methanol to fix residual biofilm for 15 min, 500 µL crystal violet dyeing solution (1%) was added and dyed for 10 min. The residual biofilm was rinsed with PBS to remove the color. Finally, the damage of the biofilm was observed by an optical microscope. Meanwhile, anhydrous ethanol was added to each hole to dissolve the stained biofilm, and the absorbance was measured at 590 nm. The biofilm destruction rate was calculated using the following Equation (8):

(8) $$\mathrm{Destruction}\;\mathrm{ratio}=\frac{\left(Abs_{\mathrm{control}}-Abs_{\mathrm{sample}}\right)}{Abs_{\mathrm{control}}}\times 100\%$$

where *Abs*_control_ is the absorbance of the blank group, and Abs_sample_ is the absorbance of each experimental group.

The rest of the biofilm was collected and dispersed in saline by ultrasonic waves. After diluted to the appropriate proportion, 100 µL solution was evenly distributed on the agar plate and then incubated at 37 °C for 24 h.

### Hemolysis Assay

Distilled water served as the positive control group, while saline was utilized as the negative control group, respectively. Rat blood cells (200 µL) and 4 mL of substance solution were incubated at 37 °C for 4 h and centrifuged at 3000 rpm. The hemolysis was photographed and recorded, and the absorbance at 540 nm of the supernatant was measured via a UV spectrophotometer. The hemolysis rate was determined using the following Equation (9):

(9) $$\mathrm{Hemolysis}\;\%=\frac{A_{\mathrm{sample}\;-\;}A_{\mathrm{negative}}}{A_{\mathrm{positive}\;-\;}A_{\mathrm{negative}}}\;\times \;100\%$$

where *A*_sample_ is the absorbance of the sample group, *A*_negative_ is the absorbance of the negative group, and *A*_postive_ is the absorbance of the positive group.

### In Vitro Biocompatibility Evaluation

The cell morphology and activity of preosteoblast cells (MC3T3‐E1) were observed to evaluate the biocompatibility. MC3T3‐E1 cells were inoculated into 24‐well plates cells (5 × 10^3^ cell cm^−2^) and cultured for 24 h. DMEM complete culture medium without material was used as the blank control group, and material solution (1 mL, 25 µg mL^−1^) was added to each well in the experimental group. At 1, 3, and 5 days of culture, live/dead staining was performed to assess the cell viability of the material. In addition, the cell proliferation was measured by CCK8 according to the kit instructions. The OD 450 of the solution was measured by an enzyme marker (Infinite F50, TECAN).

Cell morphology staining of MC3T3‐E1: Actin‐Tracker Green‐488 (Beyotime, China) was used to stain actin at day 3. Then the nucleus was stained by DAPI, and the cell morphology was observed by fluorescent microscope.

Effect of a concentration gradient of Fe_3_O_4_/CuS/Emo on cells: MC3T3‐E1 cells were inoculated into 24‐well plates cells (3 × 10^4^ cell cm^−2^) and cultured overnight. Solutions of Fe_3_O_4_/CuS/Emo nanoparticles with different concentrations (0, 12.5, 25, 50, and100 µg mL^−1^) were added to the pore plates. The microwave probe was irradiated vertically for 15 min (5 W). The cell viability was determined by the CCK8 method.

Microwave therapy in the cell and bacterial co‐culture state: MC3T3‐E1 cells were planted in 24‐well plates at a density of 3 × 10^4^ cells per well and cultured overnight. Then Fe_3_O_4_/CuS/Emo nanoparticles solution with a concentration of 25 µg mL^−1^ was added to the plate, and 100 µL bacterial solution (10^8 ^CFU) was added. The mixture was irradiated vertically with a microwave probe for (5 W, 15 min). The solution was then sucked out and diluted to a suitable number to coat the plate. In addition, the cells were washed with PBS containing 5% triantibody for 3 times, and the cell viability was determined by the CCK8 method. Finally, the morphology of cells and bacteria was observed by SEM after gradient alcohol dehydration with a silicon wafer as a carrier.

### In Vitro Osteogenic Ability Evaluation

MC3T3‐E1 cells (5 × 10^3^ cell cm^−2^) were inoculated with materials in 24‐well plates. After cultured for 4 days, MC3T3‐E1 cells were cultured in an osteogenic induction medium containing disodium β‐glycerophosphonate (10 mm), ascorbic acid (0.05 mm), and dexamethasone (10 nm). At 14 days, the cells were stained with a BCIP/NBT ALP chromogenic kit. In addition, ARS staining was used to detect the formation of a mineralized matrix.

### Evaluations of the Anti‐Inflammatory Effects of Fe_3_O_4_/CuS/Emo

The experimental procedures are listed in Supporting Information.

### In Vivo Osteomyelitis Treatment

The experimental procedures are listed in Supporting Information.

### Statistical Analysis

Data from three experiments were collected and analyzed using SPSS 21.0. One‐way ANOVA was conducted to determine statistical significance (*p < 0.05, **p < 0.01, ***p < 0.001).

## Conflict of Interest

The authors declare no conflict of interest.

## Supporting information



Supporting Information

## Data Availability

The data that support the findings of this study are available from the corresponding author upon reasonable request.

## References

[1] C. Zhong , Y. Wu , H. Lin , R. Liu , Composites, Part B 2023, 249, 110428.

[2] E. A. Masters , R. P. Trombetta , K. L. d. M. Bentley , B. F. Boyce , A. L. Gill , S. R. Gill , K. Nishitani , M. Ishikawa , Y. Morita , H. Ito , S. N. Bello‐Irizarry , M. Ninomiya , J. D. Brodell Jr. , C. C. Lee , S. P. Hao , I. Oh , C. Xie , H. A. Awad , J. L. Daiss , J. R. Owen , S. L. Kates , E. M. Schwarz , G. Muthukrishnan , Bone Res. 2019, 7, 20. 31646012 10.1038/s41413-019-0061-z PMC6804538

[3] Z. Geng , Z. Cao , J. Liu , Exploration 2023, 3, 20210117. 37323620 10.1002/EXP.20210117 PMC10191045

[4] Y. Long , L. Li , T. Xu , X. Wu , Y. Gao , J. Huang , C. He , T. Ma , L. Ma , C. Cheng , C. Zhao , Nat. Commun. 2021, 12, 6143. 34686676 10.1038/s41467-021-26456-9 PMC8536674

[5] L. Wang , Q. Yang , M. Huo , D. Lu , Y. Gao , Y. Chen , H. Xu , Adv. Mater. 2021, 33, 2100150. 10.1002/adma.202100150 34146359

[6] a) J. Fu , Y. Li , Y. Zhang , Y. Liang , Y. Zheng , Z. Li , S. Zhu , C. Li , Z. Cui , S. Wu , Adv. Mater. 2021, 33, 2102926; 10.1002/adma.202102926 34396595

[7] a) X. Chu , L. Mao , O. Johnson , K. Li , J. Phan , Q. Yin , L. Li , J. Zhang , W. Chen , Y. Zhang , Nanomed.: Nanotechnol., Biol. Med. 2019, 18, 272; 10.1016/j.nano.2019.02.016 30878657

[8] L. Li , Y. Yin , S. Zhang , J. Yang , P. Li , H. Zhou , J. Li , BMEMat 2024, e12081.

[9] Z. Z. Chen , Q. Wu , W. N. Guo , M. Niu , L. F. Tan , N. Wen , L. S. Zhao , C. H. Fu , J. Yu , X. L. Ren , P. Liang , X. W. Meng , Biomaterials 2021, 276, 121016. 34274778 10.1016/j.biomaterials.2021.121016

[10] a) Y. Qiao , X. Liu , B. Li , Y. Han , Y. Zheng , K. W. K. Yeung , C. Li , Z. Cui , Y. Liang , Z. Li , S. Zhu , X. Wang , S. Wu , Nat. Commun. 2020, 11, 4446; 32895387 10.1038/s41467-020-18268-0 PMC7477539

[11] J. Ren , Y. Qiao , L. Jin , C. Mao , C. Wang , S. Wu , Y. Zheng , Z. Li , Z. Cui , H. Jiang , S. Zhu , X. Liu , Small 2024, 20, 2307406. 10.1002/smll.202307406 38009734

[12] X. Tian , L. Zhang , M. Yang , L. Bai , Y. Dai , Z. Yu , Y. Pan , Wiley Interdiscip. Rev.:Nanomed. Nanobiotechnol. 2018, 10, e1476. 10.1002/wnan.1476 28471067

[13] J. Ma , N. Li , J. Wang , Z. Liu , Y. Han , Y. Zeng , Exploration 2023, 3, 20220161. 37933283 10.1002/EXP.20220161 PMC10582616

[14] a) Z. Chen , F. Xing , P. Yu , Y. Zhou , R. Luo , M. Liu , U. Ritz , Acta Biomater. 2024, 175, 27; 38110135 10.1016/j.actbio.2023.12.023

[15] Y. Deng , Y. Gao , T. Li , S. Xiao , M. Adeli , R. D. Rodriguez , W. Geng , Q. Chen , C. Cheng , C. Zhao , ACS Nano 2023, 17, 2943. 36688804 10.1021/acsnano.2c11448

[16] a) G. Zheng , J. Zheng , L. Xiao , T. Shang , Y. Cai , Y. Li , Y. Xu , X. Chen , Y. Liu , B. Yang , ACS Omega 2021, 6, 8672; 33817529 10.1021/acsomega.1c00606 PMC8015135

[17] Y.‐Q. Xing , S.‐Y. Liu , Chin. J. Struct. Chem. 2022, 41, 2209056.

[18] a) H. Gao , G. Liu , Y. Zhu , Z. Wen , X. Liu , G. Wang , F. Li , Green Chem. Eng. 2023, 4, 433;

[19] Y. Hu , S. Li , H. Dong , L. Weng , L. Yuwen , Y. Xie , J. Yang , J. Shao , X. Song , D. Yang , L. Wang , Adv. Healthcare Mater. 2023, 12, 2300985. 10.1002/adhm.202300985 37186891

[20] J. Liu , R. S. Li , M. He , Z. Xu , L. Q. Xu , Y. Kang , P. Xue , Biomaterials 2021, 277, 121084. 34454374 10.1016/j.biomaterials.2021.121084

[21] L. Jin , H. Liu , C. Wang , C. Mao , S. Wu , Y. Zhang , Z. Li , S. Zhu , H. Jiang , Z. Cui , Y. Zheng , X. Liu , Adv. Mater. 2024, 2410917. 10.1002/adma.202410917 39344940

[22] J. Wang , H. Cheng , W. Chen , P. Han , X. Yao , B. Tang , W. Duan , P. Li , X. Wei , P. K. Chu , X. Zhang , Chem. Eng. J. 2023, 452, 139474.

[23] a) W. Xu , S. Zhu , Y. Liang , Z. Li , Z. Cui , X. Yang , A. Inoue , Sci. Rep. 2015, 5, 18125; 26648397 10.1038/srep18125 PMC4673457

[24] S. Sheng , W. Liu , K. Zhu , K. Cheng , K. Ye , G. Wang , D. Cao , J. Yan , J. Colloid Interface Sci. 2019, 536, 235. 30368095 10.1016/j.jcis.2018.10.060

[25] a) J. Luo , H. Guo , J. Zhou , F. Guo , G. Liu , G. Hao , W. Jiang , Chem. Eng. J. 2022, 429, 132238;

[26] a) Y. Su , X. Zhang , Y. Wei , Y. Gu , H. Xu , Z. Liao , L. Zhao , J. Du , Y. Hu , X. Lian , W. Chen , Y. Deng , D. Huang , ACS Appl. Mater. Interfaces 2023, 15, 6354; 36692869 10.1021/acsami.2c17366

[27] F.‐h. Lin , R.‐a. Doong , J. Colloid Interface Sci. 2014, 417, 325. 24407694 10.1016/j.jcis.2013.11.069

[28] P. Li , J. Bi , J. Liu , Y. Wang , X. Kang , X. Sun , J. Zhang , Z. Liu , Q. Zhu , B. Han , J. Am. Chem. Soc. 2023, 145, 4675. 36800322 10.1021/jacs.2c12743

[29] X. Guo , W. Luo , L. Wu , L. Zhang , Y. Chen , T. Li , H. Li , W. Zhang , Y. Liu , J. Zheng , Y. Wang , Adv. Sci. 2024, 11, 2403388. 10.1002/advs.202403388 PMC11425287 39033533

[30] C. Papuc , G. V. Goran , C. N. Predescu , V. Nicorescu , G. Stefan , Compr. Rev. Food Sci. Food Saf. 2017, 16, 1243. 33371586 10.1111/1541-4337.12298

[31] Z. W. Liang , Y. H. Wang , J. P. Wang , T. Xu , S. L. Ma , Q. Liu , L. Q. Zhao , Y. Wei , X. J. Lian , D. Huang , Colloid. Surface. B. 2023, 227, 113358. 10.1016/j.colsurfb.2023.113358 37207386

[32] Y. Wang , S. Ma , X. Liu , Y. Wei , H. Xu , Z. Liang , Y. Hu , X. Lian , D. Huang , Colloid. Surface. B. 2023, 222, 113071. 10.1016/j.colsurfb.2022.113071 36473370

[33] a) G. Zhao , H. Lv , Y. Zhou , X. Zheng , C. Wu , C. Xu , ACS Appl. Mater. Interfaces 2018, 10, 42925; 30421911 10.1021/acsami.8b16727

[34] a) Z. Wu , H.‐W. Cheng , C. Jin , B. Yang , C. Xu , K. Pei , H. Zhang , Z. Yang , R. Che , Adv. Mater. 2022, 34, 2107538; 10.1002/adma.202107538 34755916

[35] L. Yan , X. Wang , S. Zhao , Y. Li , Z. Gao , B. Zhang , M. Cao , Y. Qin , ACS Appl. Mater. Interfaces 2017, 9, 11116. 28266215 10.1021/acsami.6b16864

[36] M. Qin , L. Zhang , H. Wu , Adv. Sci. 2022, 9, 2105553. 10.1002/advs.202105553 PMC8981909 35128836

[37] M. Yu , S. Li , X. Ren , N. Liu , W. Guo , J. Xue , L. Tan , C. Fu , Q. Wu , M. Niu , Y. Du , X. Meng , ACS Nano 2024, 18, 3636. 38227493 10.1021/acsnano.3c11433

[38] a) Q. Chen , C. Liang , X. Q. Sun , J. W. Chen , Z. J. Yang , H. Zhao , L. Z. Feng , Z. Liu , Proc. Natl. Acad. Sci. USA 2017, 114, 5343; 28484000 10.1073/pnas.1701976114 PMC5448233

[39] a) K. Kiruthiga , P. Aravindan , S. Anandan , P. Maruthamuthu , Res. Chem. Intermed. 2006, 32, 115;

[40] a) M. Xu , Y. Hu , Y. Xiao , Y. Zhang , K. Sun , T. Wu , N. Lv , W. Wang , W. Ding , F. Li , B. Qiu , J. Li , ACS Appl. Mater. Interfaces 2020, 12, 50260; 33108154 10.1021/acsami.0c14451

[41] a) W. Sun , C. Wang , D. Wan , Y. Zheng , S. Wu , J. Shen , Y. Zhang , X. Liu , Small Methods 2023, 7, 2300203; 10.1002/smtd.202300203 37116093

[42] P. Ayaz , B. Xu , X. Zhang , J. Wang , D. Yu , J. Wu , Appl. Surf. Sci. 2020, 527, 146806.

[43] B. Li , J. Mao , J. Wu , K. Mao , Y. Jia , F. Chen , J. Liu , Small 2023, 20, 2306135. 10.1002/smll.202306135 37803439

[44] a) I. Burghardt , F. Luethen , C. Prinz , B. Kreikemeyer , C. Zietz , H.‐G. Neumann , J. Rychly , Biomaterials 2015, 44, 36; 25617124 10.1016/j.biomaterials.2014.12.022

[45] a) L. Jin , H. Liu , C. Wang , X. Liu , C. Mao , Y. Zhang , Z. Li , S. Zhu , H. Jiang , Z. Cui , Y. Zheng , S. Wu , Small 2024, 2407113; 10.1002/smll.202407113 39420683

[46] L. Vigh , P. V. Escribá , A. Sonnleitner , M. Sonnleitner , S. Piotto , B. Maresca , I. Horváth , J. L. Harwood , Prog. Lipid Res. 2005, 44, 303. 16214218 10.1016/j.plipres.2005.08.001

[47] a) W. Dai , R. Shu , F. Yang , B. Li , H. M. Johnson , S. Yu , H. Yang , Y. K. Chan , W. Yang , D. Bai , Y. Deng , Adv. Mater. 2024, 36, 2305277; 10.1002/adma.202305277 37526952

[48] Z. Lin , Z. Chen , Y. Chen , N. Yang , J. Shi , Z. Tang , C. Zhang , H. Lin , J. Yin , Exploration 2023, 3, 20220149. 37933236 10.1002/EXP.20220149 PMC10624372

[49] a) S. C. Funes , M. Rios , J. Escobar‐Vera , A. M. Kalergis , Immunology 2018, 154, 186; 29455468 10.1111/imm.12910 PMC5980179

[50] F. Huang , X. Cai , X. Hou , Y. Zhang , J. Liu , L. Yang , Y. Liu , J. Liu , Exploration 2022, 2, 20210145. 37325499 10.1002/EXP.20210145 PMC10191036

[51] a) N. Kavanagh , E. J. Ryan , A. Widaa , G. Sexton , J. Fennell , S. O'Rourke , K. C. Cahill , C. J. Kearney , F. J. O'Brien , S. W. Kerrigan , Clin. Microbiol. Rev. 2018, 31; 10.1128/CMR.00084-17 PMC5967688 29444953

[52] T. Liu , M. Ma , A. Ali , Q. Liu , R. Bai , K. Zhang , Y. Guan , Y. Wang , J. Liu , H. Zhou , Nano Today 2024, 54, 102071.

