# Supplementary material for: Emodin Enhanced Microwave‐Responsive Heterojunction with Powerful Bactericidal Capacity and Immunoregulation for Curing Bacteria‐Infected Osteomyelitis
Source: Adv Sci (Weinh). 2024 Nov 27;12(3):2409979. doi: 10.1002/advs.202409979 (PMC11744657; doi:10.1002/advs.202409979)
Supplement: Supplementary file 1 — Supporting Information [file ADVS-12-2409979-s001.docx]

**Emodin Enhanced Microwave-Responsive Heterojunction with Powerful Bactericidal Capacity and Immunoregulation for Curing Bacteria-Infected Osteomyelitis**

*Tao Xu, Hao Cheng, Hailiang Pei, Jiameng Wang, Yiwei Shi, Xiangyu Zhang*, Di Huang**

Tao Xu, Hao Cheng, Hailiang Pei, Xiangyu Zhang, Di Huang

Department of Biomedical Engineering, Research Center for Nano-biomaterials & Regenerative Medicine, College of Artificial Intelligence, Taiyuan University of Technology, Taiyuan 030024, China

E-mail: huangjw2067@163.com

Hao Cheng, Jiameng Wang, Xiangyu Zhang

Shanxi Key Laboratory of Biomedical Metal Materials, College of Materials Science and Engineering, Taiyuan University of Technology, Taiyuan 030024, China

E-mail: zhangxiangyu@tyut.edu.cn

Yiwei Shi

NHC Key Laboratory of Pneumoconiosis, Department of Pulmonary and Critical Care Medicine, First Hospital of Shanxi Medical University, Taiyuan 030001, China

Figure S1. (a) SEM images of CuS. (b) SEM images of Fe_3_O_4_.


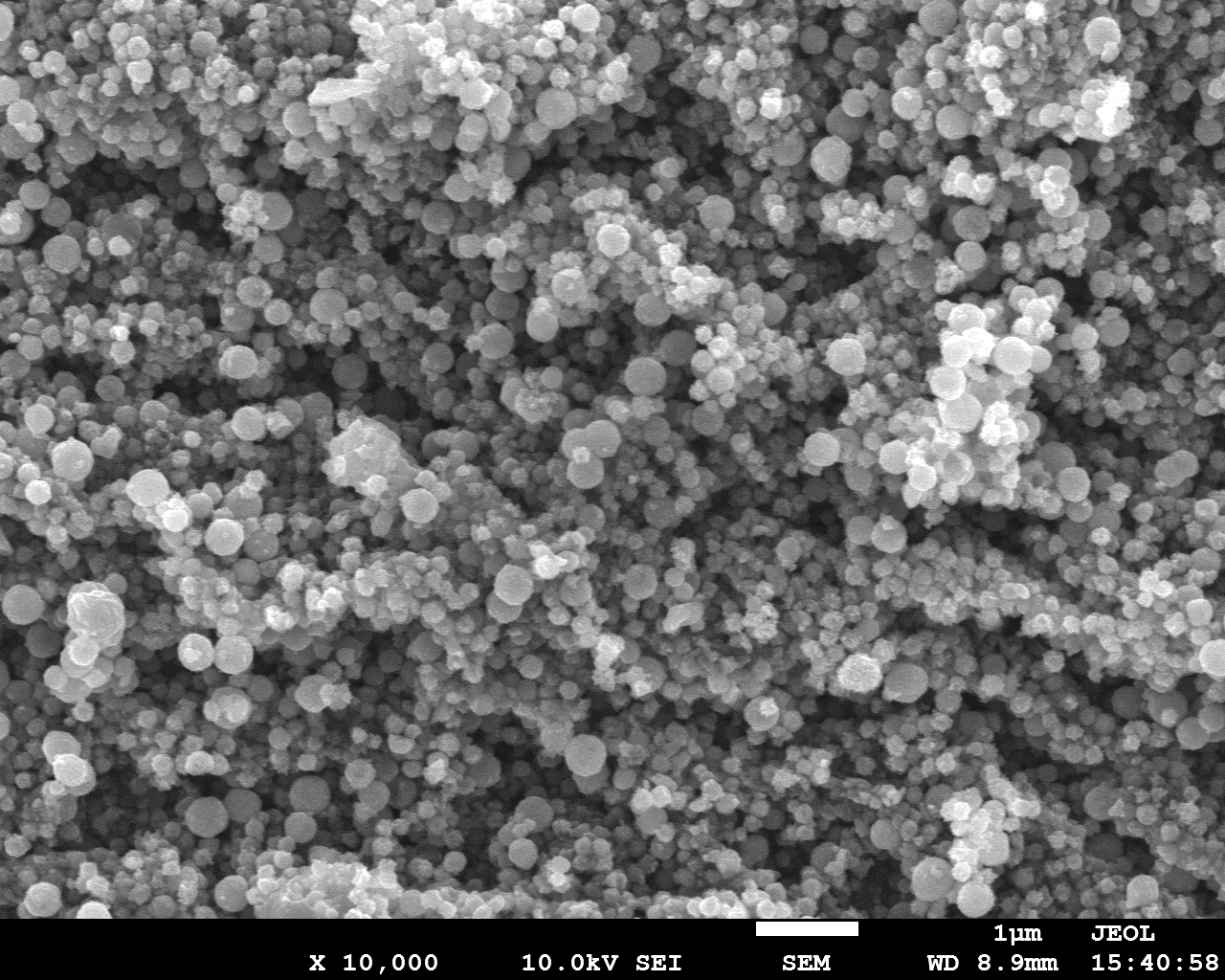

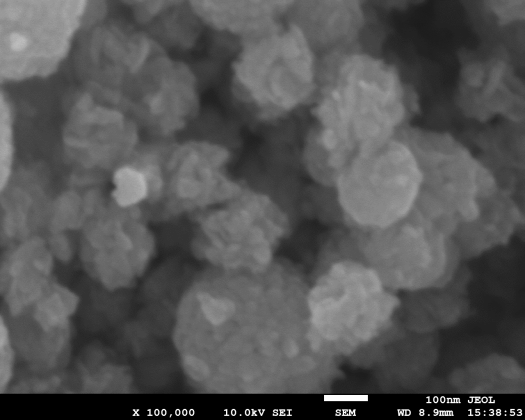


**2 μm**

**200 nm**

Figure S2. SEM images of Fe_3_O_4_/CuS.


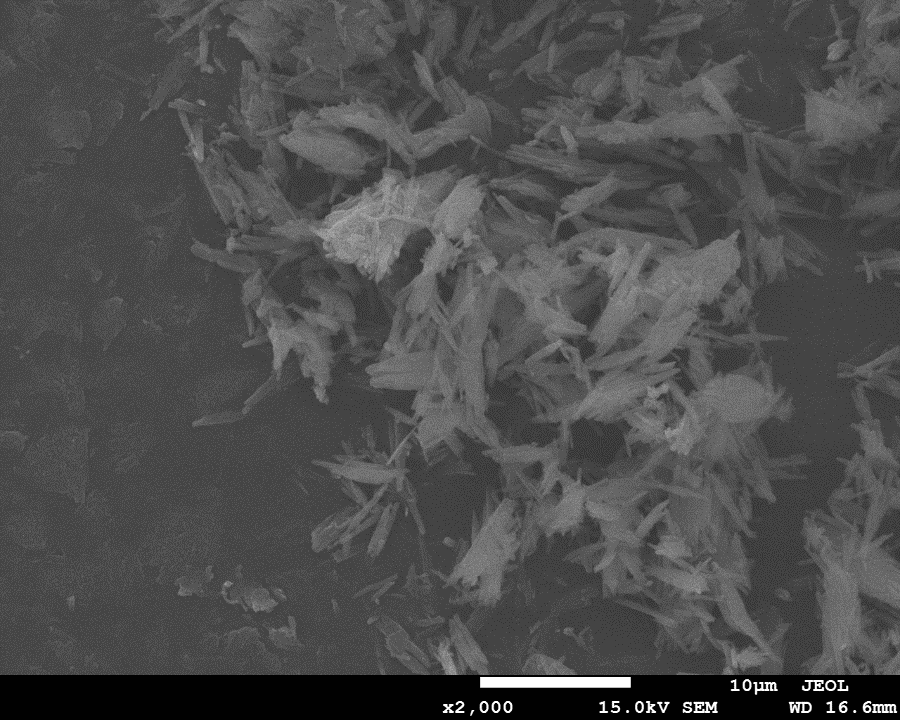


**10 μm**

Figure S3. SEM image of emodin.





Figure S4. TEM image of Fe_3_O_4_/CuS/Emo.

Figure S5. Zeta potentials of CuS, Fe_3_O_4_, Fe_3_O_4_/CuS, Emodin, and Fe_3_O_4_/CuS/Emo.


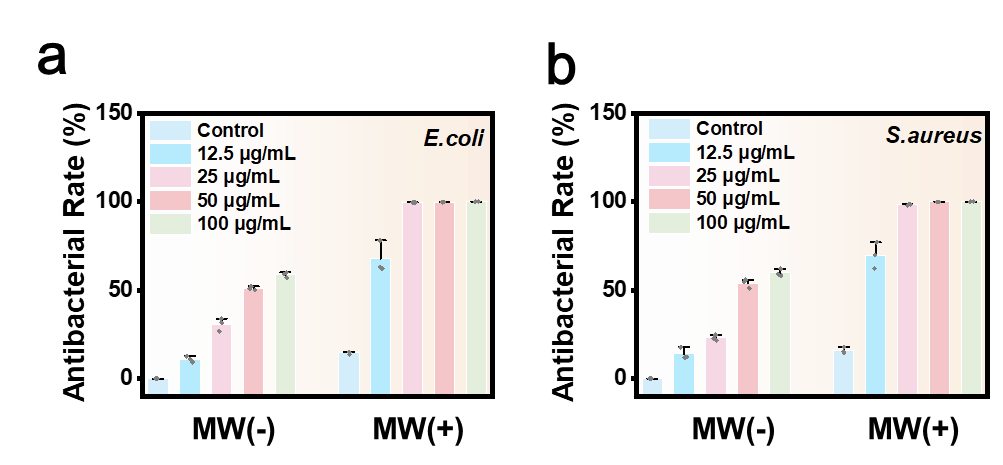


Figure S6. Antimicrobial rates of different concentrations of Fe_3_O_4_/CuS/Emo against (a) *E. coli* and (b) *S. aureus* with and without microwave treatment.

**
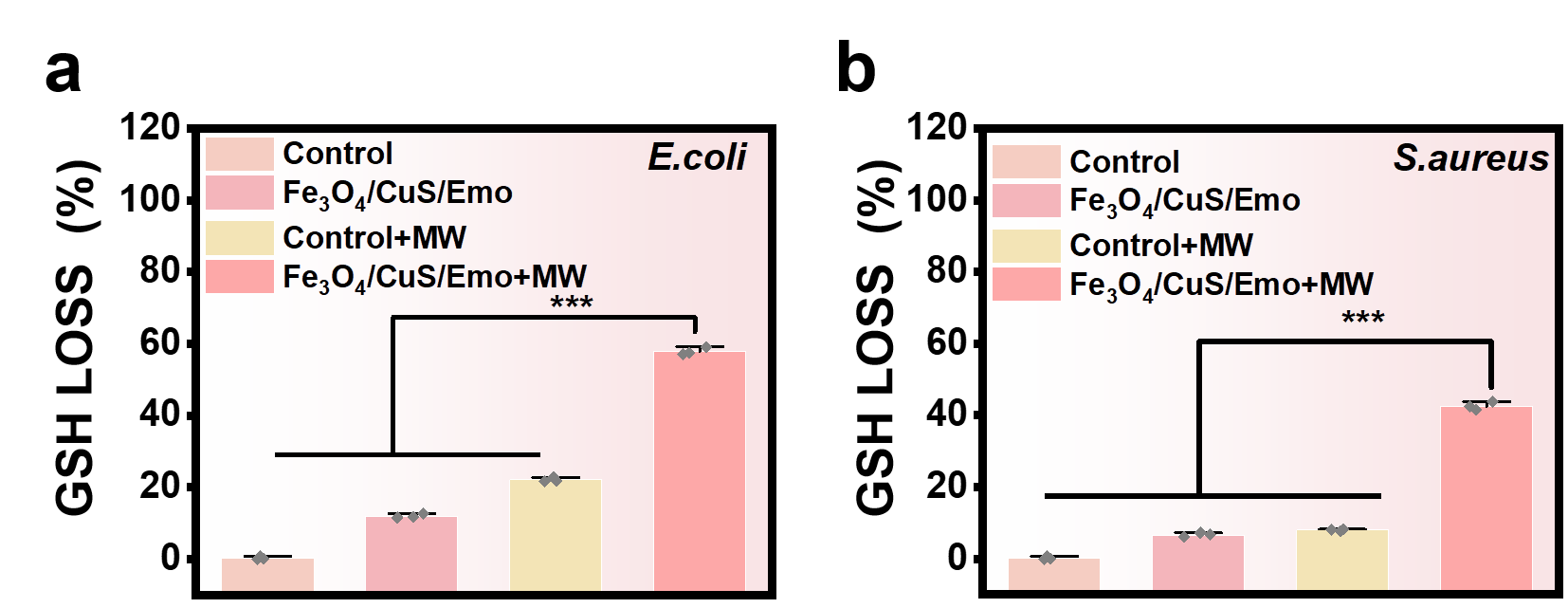
**

Figure S7. Internal GSH loss statistics of (a) *E. coli* and (b) *S. aureus* in blank group and Fe_3_O_4_/CuS/Emo group after MW treatment.


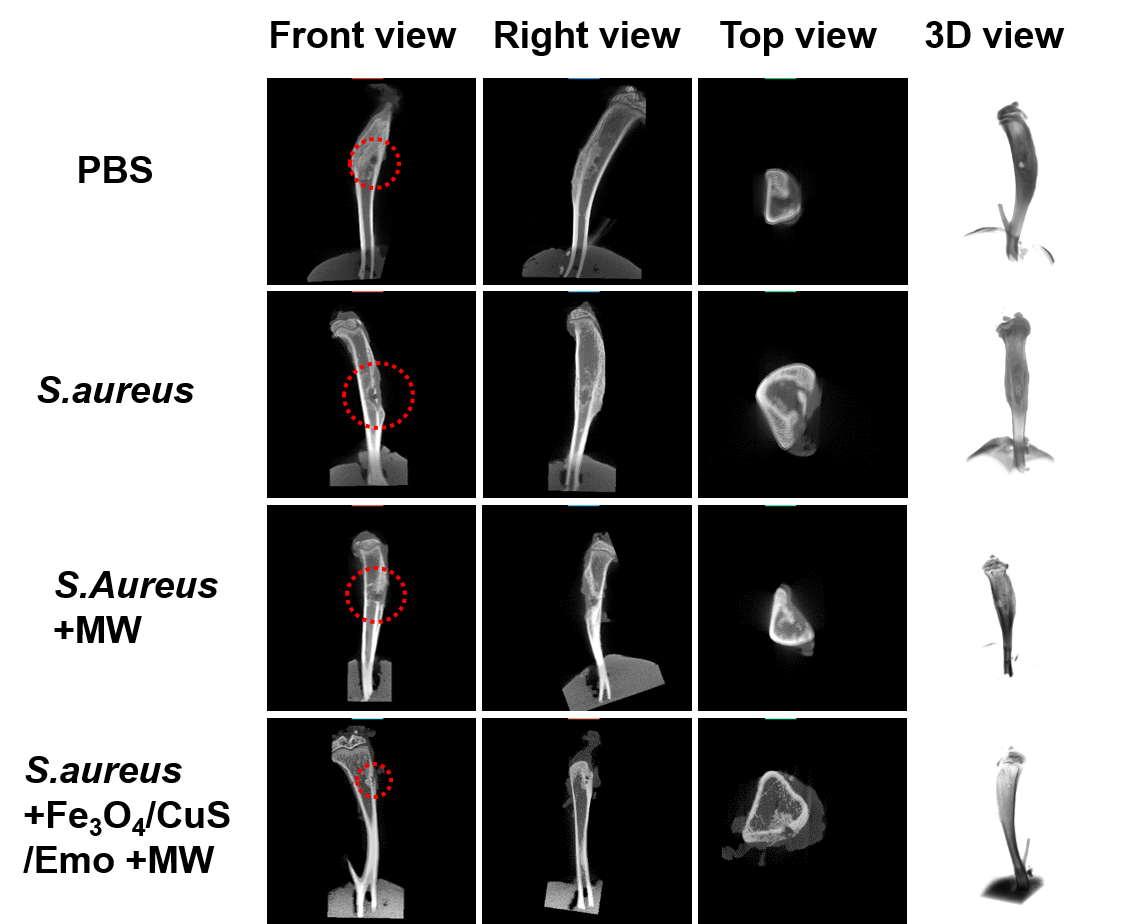


Figure S8. The micro-CT images of PBS, *S.aureus*, *S.aureus*+MW, and *S.aureus*+MW+Fe_3_O_4_/CuS/Emo groups after 14 days treatment.


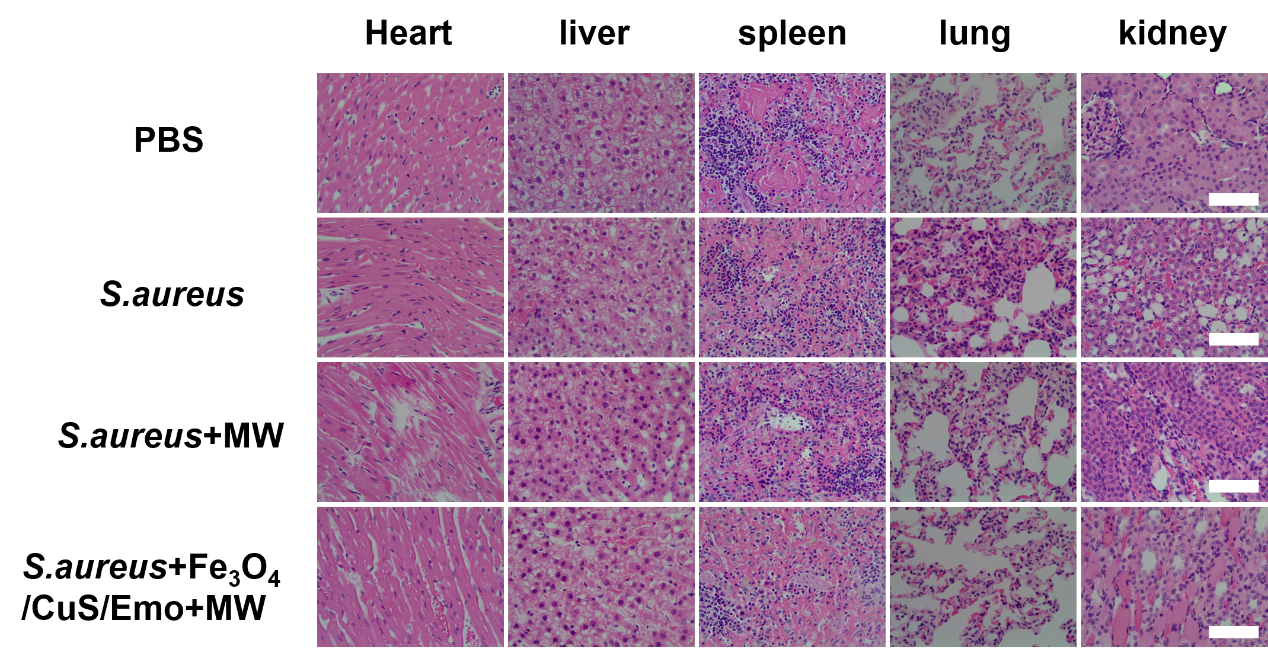


Figure S9. HE staining of heart, liver, spleen, lung and kidney in each group at day 14 (scale :100 μm).

**Experimental Section**

1. Chemicals and materials

Copper nitrate trihydrate (Cu(NO_3_)_2_⋅3H_2_O, AR), polyvinylpyrrolidone (PVP, AR), dimethyl sulfoxide (DMSO, AR)，thioacetamide (C_2_H_5_NS, AR), anhydrous sodium acetate (Na_2_SO_4_, AR), ferric chloride trihydrate (FeCl_3_·6H_2_O, AR), polyethylene glycol (PEG, AR), and polyglycol (EG, AR) were produced from Tianjin Fengboat Chemical reagent Technology Co., LTD. Hyaluronic acid (HA, 97%) was acquired from Macklin Biochemical Co. Ltd (Shanghai, China). Emodin, 3,3',5,5'-tetramethylbenzidine (TMB), Glutathione (GSH), 5,5'-dithiobide (2-nitrobenzoic acid (DNTB), o-nitrobenzene-β-D-galactoside pyranoside (ONPG) and 2',7'-Dichlorodihydrofluorescein diacetate (DFCH-DA) were purchased from Aladdin. Sirius Red (ARS), trisodium phosphate, dexamethasone, and beta-sodium glycerophosphate from Sigma-Aldrich Trading Co., LTD (Shanghai, China). Cell Counting Kit-8 (CCK8), live/dead cell staining kits, alkaline phosphatase (ALP) color development kits, 4',6-Diamidino-2 phenylindole (DAPI), BCA protein detection kits, cell lysates, alkaline phosphatase kits, lipopolysaccharide (LPS) and microfilament probes were purchased from Beyotime Biotechnology (Shanghai, China). ELISA kit was purchased from Cloud-Clone Crop. Drugs were not further purified upon receipt. The MC3T3-E1 cell line was provided by the Institute of Biochemistry and Cell Biology, Chinese Academy of Sciences (Shanghai).

**2 Materials preparation**

**2.1. Synthesis of the CuS and Fe_3_O_4_**

The CuS nanospheres were synthesized using a solvothermal method. In this process, Cu(NO_3_)_2_⋅3H_2_O (0.966 g), PVP (0.8 g), and C_2_H_5_NS (0.6 g) were combined with DMSO (60 mL) and vigorously stirred at room temperature for 0.5 h. The resulting solution was then transferred to a reactor and reacted at 120 ℃ for 20 h. Subsequently, the black precipitate was washed and centrifuged (4,000 rpm) three times with an ethanol solution. Finally, it was dried at 60 ℃ overnight.

The Fe_3_O_4_ nanospheres were prepared using a solvothermal method. Initially, FeCl_3_⋅3H_2_O (2 g) was added to EG (60 mL) and stirred thoroughly. Then, PEG (1.5 g) and CH_3_COONa (5.4 g) were added and dissolved for 0.5 h. Subsequently, the solution was transferred to a reactor and heated at 200 ℃ for 8 h. The black precipitate was washed and centrifuged (4,000 rpm) three times. Finally, it was dried at 60 ℃ for 24 h to obtain solid particles.

**2.2. Synthesis of the Fe_3_O_4_/CuS**

Fe_3_O_4_ (0.2 g) and Cu(NO_3_)_2_⋅3H_2_O (1 g) were dissolved in DMSO (40 mL). To ensure the complete adsorption of the copper ions, the mixed solution was stirred using a mechanical stirrer (1000 rpm) for 2 h. Then, C_2_H_5_NS (0.48 g) and PVP (0.64 g) were added and stirred for 1 h. Subsequently, the solution was transformed to the reactor at 120 ℃ for 20 h. Afterwards, the precipitate was washed and centrifuged (4,000 rpm) three times with anhydrous ethanol, and finally dried at 60 ℃ to obtain the Fe_3_O_4_/CuS.

**2.3. Synthesis of the** **Fe_3_O_4_/CuS/Emo**

HA (0.4 g) was added to deionized water (100 mL) through vigorous stirring until completely dissolved. Following that, Fe_3_O_4_/CuS (1.6 g) were added to the solution and mechanically stirred at a rate of 1000 rpm for 8 h. The solution was then completely frozen and finally freeze-dried for 48 h to obtain a black solid, referred to as Fe_3_O_4_/CuS/HA.

**2.4 Synthesis of the Fe_3_O_4_/CuS/Emo**

Fe_3_O_4_/CuS/HA (1 g) was redissolved in deionized water (60 mL). They were continually agitated with a mechanical stirrer to ensure total dissolution. The solution (20 mL) containing 0.15 g emodin was agitated for 8 h. The solution was then totally centrifuged and dried, yielding a final solid known as Fe_3_O_4_/CuS/Emo.

**4.15 Evaluations of the anti-inflammatory effects of Fe_3_O_4_/CuS/Emo**

To investigate the anti-inflammatory effects of Fe_3_O_4_/CuS/Emo with and without microwave treatment, an LPS-induced inflammation model was established. RAW 264.7 cells (2 × 10^4^ cell/cm^2)^ were inoculated in 24-well plates. The groups cultured for 12 h with 0.5 μg/mL LPS were used as the experimental groups. After that, the cells were co-cultured with normal medium, medium containing Fe_3_O_4/_CuS/Emo, and medium containing Fe_3_O_4_/CuS/Emo (after microwave treatment) for 24 h, respectively. The culture solution was discarded and the cells were stained with DFCH-DA probe for 30 min. In addition, after two weeks of co-culture, the macrophage surface characteristic markers TNF-α (M1-type marker) and CD206 (M2-type marker) were characterized by immunofluorescence staining. RAW 264.7 cells were fixed with 4% paraformaldehyde (Beyotime, China), and permeabilized with 0.5% Triton X-100 (Beyotime, China) and normal goat serum (New Bioscience, China) overnight. After that, they were incubated with CD206 (Beyotime, China) or TNF-α (Beyotime, China) antibodies at 4 °C overnight. Then, the cells were stained with Alexa Fluor 488 (Beyotime, China) coupled goat anti-rabbit IgG or Alexa Fluor 555 (Beyotime, China) coupled goat anti-rabbit IgG for 1 h. Finally, DAPI was used to stain the nuclei, and the stained cells were observed by confocal microscope.

Macrophage cytoskeleton staining: After the fixation and permeation of macrophages, F4/80 (macrophage marker) antibody was added and incubated at 4 ℃ overnight. They were dyed with Alexa Fluor 488 (Beyotime, China) for 1 h. The cytoskeleton was then stained with Actin-Tracker Red-555 (Beyotime, China) and DAPI was used to stain the nuclei.

Real Time PCR: Total RNA was isolated from RAW 264.7 cells utilizing ExTrizol Reagent (Protein Biotechnology, PR90). This total RNA underwent reverse transcription to cDNA via PrimeScript™ RT. The process incorporated the gDNA Eraser (RR047A, Takara) before the reverse transcription to cDNA. Subsequently, the gene expression level was analyzed with the TB Green® Premix Ex Taq™ (RR047A, RR420A, Takara) in a StepOne real-time PCR system The primer sequence was as follows:

GAPDH-G:TCA ACG GCA CAG TCA AGG

GAPDH-R: TTA GTG GGG TCT CGC TCC

TNF-A-G:CAG GCG GTG CCT ATG TCT C

TNF-A-R: CGA TCA CCC CGA AGT TCA GTA G

CD206-F: TGT ACG CAG TGG TTG GCA GTG

CD206-R: GCT CTG ATG ATG GAT TTC CTG GTA G

To normalize mRNA expression levels, GAPDH mRNA expression was used as a reference. The results were derived through the comparative cycle threshold (ΔΔCt) method.

**4.16 In vivo Osteomyelitis Treatment**

The study was approved by the International Animal Welfare Standards Animal Ethics Committee and strictly performed in accordance with the guidelines for the care and use of laboratory animals of Taiyuan University of Technology (approval number: TYUT2024041705). Healthy adult male SD rats (250 g) were firstly divided into four groups: PBS group, *S. aureus* group, *S. aureus*+MW group and *S. aureus* +Fe_3_O_4_/CuS/Emo+MW group. During modeling of osteomyelitis, the skin was cut longitudinally from the edge of the tibia to expose the tibia, and then a hole was drilled with a dental drill. Then 50 μL of PBS or *S. aureus* solution (about 10^7^ CFU/mL) was injected into the bone marrow cavity at day -1. At day 0, 50 μL of Fe_3_O_4_/CuS/Emo (25 μg/mL) was injected into the bone marrow cavity. The pores were sealed with bone wax and the wounds were closed with surgical sutures. Similar to the MW heating experiment *in vitro*, the surgery sites of the tibia in *S. aureus*+MW group and *S. aureus* +Fe_3_O_4_/CuS/Emo+MW group were irradiated with microwave for 10 min at day 1 to 3. During this process, the temperature was kept at around 50 ℃. Rats were euthanized at 7 and 14 days of culture. After the tibiae were removed, the bone marrow fluid inside was coated with plate and rye stained. Section staining of bone tissue was performed by Wuhan Boerfu Biotechnology Co.,Ltd.
